# Evolution of Exenatide as a Diabetes Therapeutic

**DOI:** 10.2174/1573399811309020007

**Published:** 2013-03

**Authors:** Sunil Bhavsar, Sunder Mudaliar, Alan Cherrington

**Affiliations:** 1 Amylin Pharmaceuticals, Inc., San Diego CA; 2 VA San Diego Healthcare System and University of California at San Diego, San Diego CA; 3 Vanderbilt University, Nashville TN, USA

**Keywords:** Diabetes mellitus, Exenatide, Exendin-4, GLP-1 receptor agonist.

## Abstract

Type 2 diabetes (T2DM) is a disease of epidemic proportion associated with significant morbidity and excess mortality. Optimal glucose control reduces the risk of microvascular and possibly macrovascular complications due to diabetes. However, glycemic control is rarely optimal and several therapeutic interventions for the treatment of diabetes cause hypoglycemia and weight gain; some may exacerbate cardiovascular risk. Exenatide (synthetic exendin-4) is a glucagon-like peptide-1 receptor (GLP-1R) agonist developed as a first-in-class diabetes therapy. This review presents an overview of the evolution of exenatide as a T2DM treatment, beginning with the seminal preclinical discoveries and continuing through to clinical pharmacology investigations and phase 3 clinical trials. In patients with T2DM, exenatide enhanced glucose-dependent insulin secretion, suppressed inappropriately elevated glucagon secretion, slowed gastric emptying, and enhanced satiety. In controlled phase 3 clinical trials ranging from 12 to 52 weeks, 10-mcg exenatide twice daily (ExBID) reduced mean HbA_1c_ by -0.8% to -1.7% as monotherapy or in combination with metformin (MET), sulfonylureas (SFU), and/or thiazolidinediones (TZD); with mean weight losses of -1.2 kg to -8.0 kg. In controlled phase 3 trials ranging from 24 to 30 weeks, a 2-mg once-weekly exenatide formulation (ExQW) reduced mean HbA_1c_ by -1.3% to -1.9%, with mean weight reductions of -2.3 to -3.7 kg. Exenatide was generally well-tolerated. The most common side effects were gastrointestinal in nature, mild, and transient. Nausea was the most prevalent adverse event. The incidence of hypoglycemia was generally low. By building upon early observations exenatide was successfully developed into an effective diabetes therapy.

## INTRODUCTION

Diabetes is a disease of epidemic proportion [1,2], but glycemic control is rarely optimized and some therapeutic interventions cause hypoglycemia, weight gain and possibly exacerbate cardiovascular (CVD) risk [3]. Data from large studies indicate that restoration of glycemic control when coupled with weight loss can improve hypertension, dyslipidemia, and other CVD risk markers [4-6]. 

**Fig. (1):**
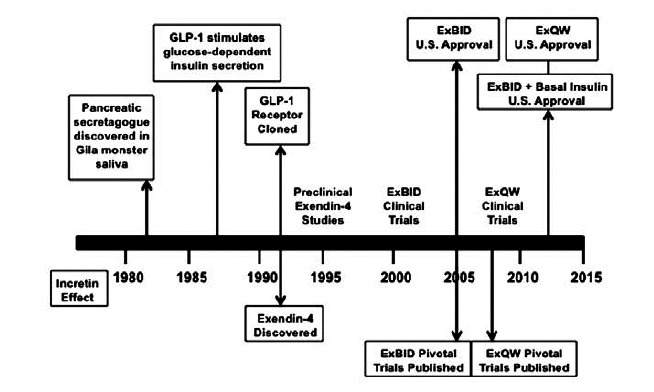
Timeline for the evolution of exenatide (synthetic exendin-4) as a diabetes therapeutic. (Created from references [7,12,13,16-24,32-34,39,40-57,60-66,69-121,126,127,130-132,135,137,145]).

Exenatide (synthetic exendin-4) is a glucagon-like peptide-1 (GLP-1) receptor agonist that has been developed as a first-in-class diabetes therapy. This review presents an overview of the evolution of exenatide as a treatment for type 2 diabetes (T2DM) from seminal preclinical discoveries, clinical pharmacology investigations, and phase 3 clinical trials (Fig. **1**). By building upon early observations of antihyperglycemic actions by GLP-1 and Gila monster exendin-4, exenatide was successfully developed into a diabetes therapeutic agent with a broad range of beneficial cardiometabolic effects and an acceptable tolerability profile. A twice daily (BID) exenatide formulation is currently approved for use in patients with T2DM in the U.S., E.U., Japan, and other countries. A once weekly (QW) exenatide formulation is also currently approved for use in the U.S., E.U., and Japan, while a once-monthly formulation is undergoing clinical testing.

## INCRETINS 

**Fig. (2):**
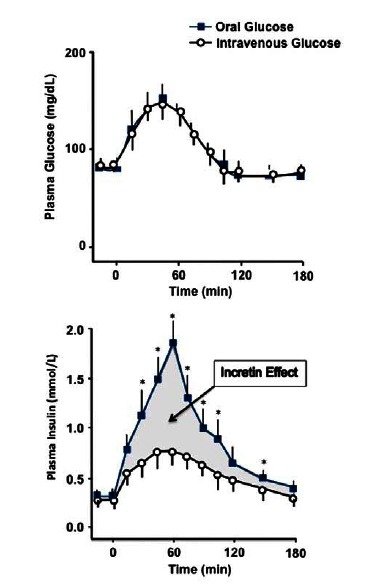
**(A)** Representative diagram of the incretin effect in healthy volunteers after administration of a 50 g oral glucose bolus or an equivalent isoglycemic intravenous glucose infusion. The increasing plasma glucose concentrations (*left panel*) results in an increase of plasma insulin concentrations (*right panel*). However, the route of glucose administration (oral or intravenous) determines the extent of the resulting β-cell response. This difference (incretin effect) prompted investigations into the role of incretins, factors secreted from the intestinal tract upon food ingestion that enhance the insulin secretion. (Adapted from sub in reference [8]). (**B) **Illustration depicting the normal physiological actions of GLP-1. GLP-1 stimulates replenishment of insulin stores via stimulation of proinsulin gene expression in β-cells; promotes the differentiation of progenitors derived from pancreatic islets into functioning β-cells; reduces apoptosis in pancreatic islets; and reduces ambient glucose concentrations via inhibition of glucagon secretion from pancreatic α-cells. GLP-1 also decelerates the rate of gastric emptying; reduces food intake and body weight; and promotes satiety. In addition, GLP-1 acts on the heart by improving myocardial function and cardiac output. (Adpated from references [154] and [155]).

The 'incretin effect' (Fig. **2A**) was originally described as the observation that oral glucose stimulates insulin secretion to a greater extent than an intravenous infusion of glucose [7-11]. Later, ingestion of various solid foods was also found to stimulate this physiological response to nutrient ingestion. GLP-1 and glucose-dependent insulinotrophic polypeptide (ie, gastric inhibitory polypeptide [GIP]) are the gut hormones primarily responsible for the incretin effect. In healthy, normoglycemic adults the contributions of GLP-1 and GIP to the insulin secretory response to elevated circulating glucose concentrations (nutrient ingestion) are estimated to be 73% of the total response. Because of its pleiotropic metabolic effects and for the reasons explained below, GLP-1 quickly became the primary focus of drug development efforts for treating diabetes (Fig. **2B**).

### The GLP-1 Receptor and GLP-1

The GLP-1 receptor (GLP-1R) is a seven-transmembrane domain, G-protein coupled receptor expressed in pancreatic periductal- and β-cells, kidney, heart and blood vessels, stomach, small and large intestine, lung, and brain [12-16] The physiology of the GLP-1R and its ligand have been extensively explored within the context of glucose homeostasis using GLP-1R(-/-) knockout (KO) mice [17-24]. GLP-1R KO mice have elevated blood glucose concentrations following an oral glucose challenge, coupled with lower than normal plasma insulin concentrations [17,19,20]. GLP-1 fails to stimulate insulin release from isolated pancreatic islets from GLP-1R KO mice, in sharp contrast to the 2-fold insulin stimulation in islets from normal mice and despite equivalent insulin content in islets from both mouse strains [18]. Similar results were observed using perfused pancreata [19]. In addition, De León *et al.* [24] examined β-cell mass regeneration after GLP-1R KO mice were 70% pancreatectomized. Five weeks post-surgery, β-cell mass was not significantly different between wild-type pancreatectomized or sham-operated mice. However, in GLP-1R KO mice, β-cell mass remained significantly lower in pancreatectomized versus sham-operated animals, confirming a role for GLP-1 in pancreatic islet regeneration in the mouse. 

GLP-1, the only endogenous ligand for the GLP-1R, is rapidly secreted by the L-cells of the intestinal mucosa in response to nutrient ingestion [25]. This peptide hormone enhances glucose-dependent insulin secretion, suppresses inappropriately elevated glucagon secretion, slows gastric emptying, and reduces food intake [25-31]. In rodent models, GLP-1 treatment is associated with pancreatic islet neogenesis, β-cell proliferation, and increased β-cell mass [32-34]. 

**Fig. (3):**
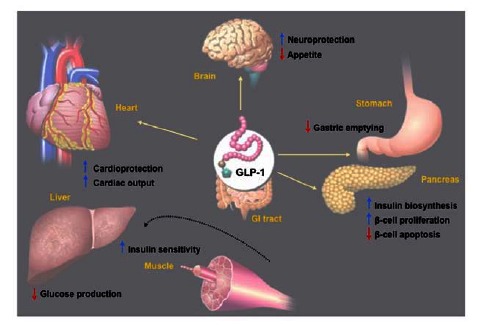
Treatment effects of continuous subcutaneous GLP1 infusion for 6 weeks in patients with T2DM. **(A, B)** Plasma glucose concentrations. GLP-1 significantly reduced fasting plasma glucose compared with saline or baseline.** (C, D)** Plasma C-peptide concentrations during a hyperglycemic clamp. The insert shows the first-phase secretory response (incremental 0-10 min) for patients receiving GLP-1. GLP-1 significantly stimulated the β-cell secretory response compared with saline or baseline. Mean±SEM. (Reprinted with permission from reference [**35**].)

Initially, a role for GLP-1 in ameliorating diabetes pathophysiology was explored using continuous infusion techniques to increase circulating GLP-1 concentrations [35]. In patients with T2DM, 6 weeks of continuous GLP-1 infusion significantly reduced HbA_1c_, fasting glucose, and post-prandial glucose excursions compared with placebo (PBO) infusion (Fig. **3**). GLP-1 infusion also improved β-cell sensitivity to ambient glucose by 77% compared with baseline, and re-established a more normal pattern of both first- and second-phase insulin secretion. Gastric emptying was slowed and patients lost weight. GLP-1 reduced sensations of hunger, i.e., enhanced satiety and fullness, and reduced prospective food intake; these effects were absent with PBO infusion. In another study, intravenous infusion of GLP-1 prior to, and during, a glucose challenge in patients with T2DM was associated with an insulinotropic effect on both first- and second-phase insulin secretion, augmentation of glucose disappearance rates, and suppression of circulating glucagon levels [36]. Despite these positive effects, GLP-1 augmentation as a strategy to treat diabetes was deemed impractical, because GLP-1 is rapidly degraded in the circulatory system, by the enzyme dipeptidyl peptidase-IV (DPP-4), with a half-life of less than 2 minutes [37, 38]. 

## EXENDIN-4

**Fig. (4):**
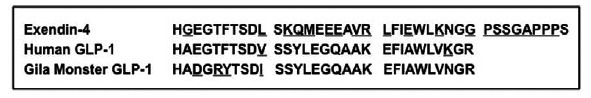
Amino acid sequence comparison for exendin-4 and GLP-1. (Adapted from references [13,40,41]).

Exendin-4, the naturally occurring form of exenatide, was investigated as a potential ligand for the GLP-1R and a possible diabetes therapy after a number of nonclinical discoveries. Exendin-4 was originally isolated from the salivary secretions of the lizard *Heloderma suspectum* (Gila monster) [39,40]. Exendin-4 has a 53% amino acid sequence homology with mammalian GLP-1 (Fig. **4**) [39,40]. However, mammalian GLP-1 is processed from the proglucagon gene in L-cells in the small intestine, whereas lizard exendin-4 is transcribed from a different gene, not the Gila monster homologue of the mammalian proglucagon [41]. The second seminal discovery was that exendin-4 is resistant to degradation by DPP-4, the enzyme responsible for rapidly degrading endogenous GLP-1 [37,38]. Third, although exendin-4 is not an analogue of GLP-1, exendin-4 and GLP-1 share many glucoregulatory actions mediated by the GLP-1 receptor [42]. The pancreatic GLP-1 receptor binds exendin-4 and GLP-1 with equal affinity *in vitro*, and both peptides stimulate the receptor equipotently [42-45]. Hence, exendin-4 is a GLP-1R agonist. 

Actions shared by exendin-4 and GLP-1 in non-human studies include reduction of hyperglycemia, glucose-dependent enhancement of insulin secretion, glucose-dependent suppression of inappropriately high glucagon secretion, slowing of gastric emptying, and reduction of food intake, often with body weight reduction or blunting of weight gain [39,44,46-49]. In both *in vitro* and *in vivo* preclinical models, exendin-4 promoted β-cell proliferation and islet neogenesis from precursor cells [33,50-56]. Further, as a result of its enhanced pharmacokinetics compared to GLP-1, exendin-4 demonstrated very high *in vivo* potency relative to native GLP-1 [44,46,56]. 

### Exendin-4 and Insulin-Secreting β-cells in Animal Models of Diabetes

The actions of exendin-4 on insulin secretion from pancreatic β-cell has been studied in a number of animal models of T2DM. T2DM is characterized by progressive loss of β-cell function [3,4,5,58,59]. The emergence of overt T2DM is precipitated by the failure of pancreatic β cells to secrete adequate amounts of insulin to compensate for insulin resistance in peripheral tissues, primarily liver and muscle. Further, T2DM is characterized by the emergence of post-prandial- and subsequently, fasting- hyperglycemia. In the liver, insulin resistance is manifested by glucose overproduction during the basal state despite fasting hyperinsulinemia, combined with impaired hepatic response to the normal elevation in postprandial insulin. In muscle, insulin resistance manifests as impaired postprandial glucose uptake. 

A number of preclinical reports demonstrated exendin-4-mediated restoration of function and mass of insulin-secreting islets in the rodent pancreas [32,50,52-56]. These models of T2DM and obesity include the obese (*ob/ob*) mouse that is deficient in the hormone leptin and expresses insulin resistance and impaired insulin tolerance, the *db/db* mouse that has a defect in leptin signaling and expresses insulin resistance, hypertriglyceridemia and has impaired glucose tolerance, the partial-pancreatectomized rodent model (rats or mice), the Goto-Kakizaki rat that is genetically predisposed to a neonatal β-cell mass deficiency, the insulin-resistant obese *fa/fa* diabetic Zucker rat, the nondiabetic insulin-resistant obese *fa/fa* Zucker rat, and spontaneously-diabetic Rhesus monkeys. For example, in mice genetically predisposed to develop diabetes (*db/db*), exendin-4 increased β-cell proliferation and islet mass, ameliorated the normal development of hyperglycemia, improved insulin secretion, and reduced HbA_1c_. Exendin-4 also blocked β-cell apoptosis, increased pancreatic expression of transcription factors, and enhanced islet neogenesis. Due to the difficulty of reproducing this type of experiment in humans, it is not known whether the same mechanism is active in patients with T2DM. 

Glucose-dependent insulinotropism, the ability of agents to stimulate insulin secretion during euglycemia or hyperglycemia, but not during hypoglycemia, was elucidated in some of the preclinical models of diabetes described above [39,44,49]. For example, in diabetic *db/db* mice, chronic exendin-4 administration resulted in fasting serum insulin concentrations notably higher than with vehicle treatment under conditions of hyperglycemia [44]. HbA_1c_ and fasting glucose concentrations were significantly reduced, but did not quite reach normoglycemic levels, with no evidence of hypoglycemia. Another preclinical study investigated whether the previously-described effects of exendin-4 on insulin sensitivity, appetite, and body weight were distinct from its antihyperglycemic effects [53]. In this study, exendin-4 increased β-cell mass to a greater extent in nondiabetic insulin-resistant obese *fa/fa* Zucker rats than would be expected in animals of comparable insulin-resistance, suggesting a direct trophic effect of exendin-4 on pancreatic islet neogenesis, independent of improvements in metabolic parameters. This study was the first to show that exendin-4 could improve insulin sensitivity in an environment of insulin resistance, partly due to reductions in food intake and partly independent of changes in body weight and ambient glycemia. 

#### Exendin-4 and Body Weight in Animal Models of Diabetes and/or Obesity

In nonclinical diabetes models, weight loss or blunted weight gain are consistently associated with exendin-4 treatments [39,46,47,48]. Studies have extended these findings to include obese, normoglycemic animal models. For example, exendin-4 treatment for 56 days reduced food intake and slowed weight gain in obese *fa/fa* Zucker rats [47]. Corresponding reductions in HbA_1c_, fasting glucose, and fasting insulin also occurred. In a later study, acute exendin-4 administration dose-dependently suppressed food intake in normoglycemic mice and rats fed a high fat diet, while chronic, continuous exendin-4 infusion significantly slowed body weight gain, decreased fat mass, and spared lean tissue [48]. Food intake, total cholesterol, triglycerides and insulin levels were all significantly reduced. These data suggest that exendin-4 has an effect on metabolic pathways regulating food ingestion. 

## EXENATIDE (SYNTHETIC EXENDIN-4) CLINICAL PHARMACOLOGY AND EXPLORATORY INVESTIGATIONS

### Overview

Clinical pharmacology studies have been conducted using synthetic exendin-4 (exenatide) administered as subcutaneous injections or intravenous infusions. These studies have identified the appropriate dosing approach for clinical use, and explored the mechanisms of action in humans. Initial dosing strategies explored single bolus, continuous infusion, and twice (BID) and thrice (TID) daily subcutaneous administration before meals. In these studies, subcutaneous injections of 10-mcg exenatide twice daily (ExBID) demonstrated a consistent pattern of pharmacokinetics and pharmacodynamic responses, with transient, mild-to-moderate nausea as the primary side effect [60-65]. There was a minimal dependence of exenatide pharmacokinetic profiles on body weight, supporting development of a fixed dosage regimen for ExBID [59,62]. These studies also found utility in starting patients on a 5-mcg exenatide BID dose, generally for 4 weeks, before increasing to the maintenance dose of 10-mcg BID [66]. This graduated approach mitigated much of the nausea and vomiting observed when initiating directly with the 10-mcg BID dose.

The mechanisms of action elucidated in nonclinical experiments with exendin-4 were largely confirmed in early human studies in both healthy individuals and in patients with type 2 diabetes. In patients with T2DM, exenatide slowed gastric emptying; reduced fasting hyperglycemia and hyperglucagonemia; stimulated glucose-dependent insulin secretion (pancreatic β-cell function); attenuated post-prandial excursions of glucose, insulin, and glucagon; inhibited food intake; and reduced body weight. The integrated effects of these mechanisms are likely important for the clinical efficacy of ExBID. 

### Studies on Gastric Emptying and Satiety

The effects of exenatide on gastric emptying in patients with T2DM are best understood within the context of the important role the rate of nutrient delivery from the stomach to the small intestine plays in the extent of post-prandial glucose excursions [67,68]. One of the main confounders in understanding the pathophysiology of gastric emptying in diabetes is hyperglycemia itself, as elevated glucose concentrations in healthy individuals slow the rate of gastric emptying in order to moderate postprandial glucose excursions [67]. Thus the gastric emptying rate in the face of diabetic hyperglycemia may be considered pathophysiological, as gastric emptying is relatively accelerated in patients with diabetes, compared with the usually expected slowing of gastric emptying that occurs in healthy individuals under similar hyperglycemic conditions. In patients with T2DM (without severe autonomic neuropathy), gastric emptying rates are often accelerated compared with healthy individuals. Conversely, patients with T2DM and severe autonomic neuropathy often have reduced gastric emptying (gastroparesis).

For exenatide, slowing of gastric emptying and reduced food intake were first established as important mechanisms-of-action in nonclinical experiments [44,47]. Later, clinical pharmacology studies confirmed slowed gastric emptying in patients with T2DM given various exenatide doses including clinically-relevant doses [59-62,69,70]. Using scintigraphy, 5-days of ExBID was shown to slow gastric emptying of both the solid and the liquid components of a meal [70]. The T_50_ (time to achieve 50% stomach emptying) for solid meal components was 169 minutes, significantly slower than the T_50_ of 60 minutes for PBO. The T_50_ for liquid meal components was 114 minutes, also significantly slower than the T_50_ of 34 minutes for PBO. Thus, ExBID increased the T_50 _3-fold for both solids and liquids compared with PBO. 

Clinical studies have also documented a significant reduction of food intake and patient reports of increased satiety after exenatide administration in both normo- and hyperglycemic individuals [69,71,72]. In lean (mean BMI 25 kg/m^2^), healthy young adults, bolus injections of 10 mcg exenatide reduced caloric intake, overall food intake, and ingestion of proteins, carbohydrates, and total fat, compared with placebo [71]. When exenatide was injected 60 minutes prior to a standardized meal, mean caloric intake was significantly lower (463 kcal compared with 672 kcal for placebo). Post-prandial glucose concentrations at 60 and 120 minutes after meals were also reduced. In another study enrolling healthy volunteers, exenatide acutely reduced food intake and slowed gastric emptying [72]. Subjects consumed 19% fewer calories at a free-choice buffet lunch with exenatide than with placebo (867±79 kcal versus 1,075±93 kcal, respectively; p=0.012). Further, T2DM patients administered exenatide had significantly reductions in caloric intake, postprandial triglycerides, and body weight, compared to treatment with the DPP-4 inhibitor sitagliptin [69]. Exenatide reduced total caloric intake by -134±97 kcal compared with an increase of +130±97 kcal with sitagliptin.

### Integrated Effects of Exenatide on Glycemia

**Fig. (5):**
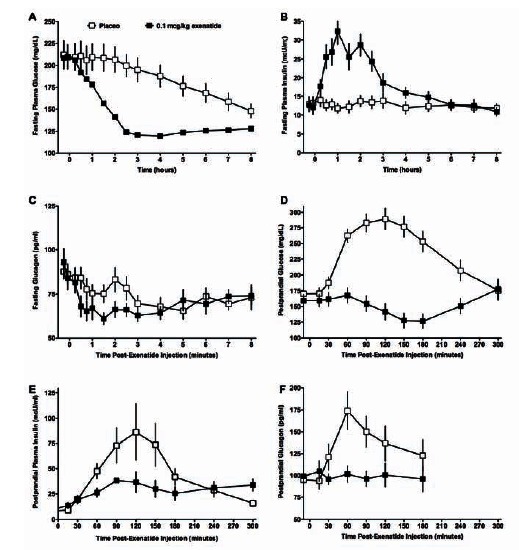
Exenatide treatment for 5 days restored a more normal glycemic profile in patients with T2DM. Fasting plasma glucose (**A**), serum insulin (**B**), and (C) glucagon concentrations during the 8 hours following a single dose of placebo (n=11) or 0.1 µg/kg exenatide administered at t = 0 (n=12). Mean±SEM. (**D, E, F**) Postprandial plasma glucose (**D**), insulin (**E**), and glucagon (**F**) concentrations following a single dose of placebo or 0.1 µg/kg exenatide at t=0 followed by a standardized meal. Postprandial glucose: Placebo n=20; Exenatide n=20. Post-prandial insulin: Placebo n=16; exenatide n=16. Postprandial glucagon: Placebo n=20; Exenatide n=20. **(**Adapted from reference [**61**]).

In patients with T2DM, exenatide rapidly lowers both fasting and postprandial plasma glucose by multiple mechanisms [60-61,69,72,74]. In the postprandial state, exenatide slows gastric emptying to modulate the rate of nutrient absorption and moderate the extent of postprandial glucose excursions. Representative dose-response profiles found during these early exenatide clinical studies for the simultaneous changes in glucose, insulin, and glucagon occurring after an exenatide injection demonstrated these actions at clinically-relevant doses (Fig. **5**). In two separate early-stage, 28-day clinical trials, exenatide also dose-dependently reduced HbA_1c_ [60,63]. 

Two other clinical pharmacology studies investigated the relative contributions of slowed gastric emptying, enhanced insulin secretion, glucagon suppression, and splanchnic and peripheral glucose metabolism on postprandial glucose excursions in patients with T2DM after intravenous exenatide administration [75,76]. Glucose retained within the splanchnic bed consists of glucose taken-up and stored by the liver, glucose metabolized by gastrointestinal tissues, and glucose remaining in the gastrointestinal tract. Infusion of exenatide with an oral glucose bolus or a mixed meal attenuated the rate of glucose appearance in the circulatory system. Exenatide decreased endogenous glucose release from the liver by approximately 45% and increased glucose retention in the splanchnic area. However, exenatide did not increase tissue sensitivity to insulin, nor did it alter the rate of total body glucose disposal. 

In multiple clinical pharmacology studies in patients with T2DM, exenatide was shown to modulate fasting serum insulin and glucagon in a manner dependent upon exenatide dose and ambient glucose concentrations [60-62,64,69,77-80]. Both acute and chronic exenatide therapy improved β-cell secretory function (i.e., insulin secretion in response to hyperglycemia). In patients with T2DM with a blunted first phase insulin secretory response after acute elevation of blood glucose levels, intravenous exenatide in the therapeutic range restored the normal first phase insulin secretory response [79]. Exenatide also augmented the second-phase insulin secretory response. Of note, the overweight patients with T2DM enrolled in this study had a mean HbA_1c_ of 6.6%, indicating good glycemic control at baseline. 

The ability of exenatide to dose-dependently suppress inappropriately-elevated glucagon secretion was demonstrated in patients with T2DM in a number of clinical pharmacology studies [61,62,63,69,80]. Exenatide-mediated reductions in plasma glucagon concentrations under fasting conditions support the hypothesis that suppression of glucagon secretion is not merely a consequence of the slowing of nutrient presentation to the small intestine (gastric emptying). 

**Fig. (6):**
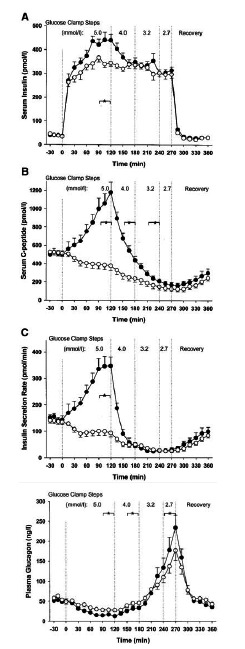
Insulin secretory response and glucagon counterregulatory hormone response. Basal period: ~30 to 0 min. Infusion of exenatide or placebo commenced at 0 min. Glycemic steps: 0-120 min, euglycemia with plasma glucose at ~5.0 mmol/L; 120–180 min, hypoglycemia with plasma glucose at ~4.0 mmol/L; 180-240 min, hypoglycemia with plasma glucose at ~3.2 mmol/L ending in nadir of ~2.8 mmol/L; 270-360 min, recovery phase. Open circles, placebo treatment arm; Filled circles, exenatide treatment arm. Data are mean±SEM. N=11 per treatment arm. *P<0.05, exenatide versus placebo during the steady-state of the indicated glycemic interval. (Reprinted with permission from reference [82]).

Degn *et al.* [81] explored glucose-dependent insulin secretion and the counter-regulatory response to hypoglycemia in healthy volunteers given an intravenous infusion of exenatide or PBO (Fig. **6**). In the presence of euglycemic hyperinsulinemia, insulin secretory rates with exenatide were significantly (3.5-fold) higher than with PBO. During hypoglycemia, insulin secretory rates declined similarly and rapidly in both groups. Glucagon was markedly more suppressed with exenatide than with PBO (~50% lower) under conditions of euglycemia. During worsening of hypoglycemia, glucagon increased progressively with both treatments, but to a significantly greater extent with exenatide. Plasma glucose recovery time was equivalent for both treatments. During the recovery interval, glucagon response was equivalent in both treatment arms. Circulating levels of cortisol, epinephrine, norepinephrine, and growth hormone were similar between treatment arms, indicating preservation of the counter-regulatory response to hypoglycemia during exenatide exposure. 

### Effects of Exenatide on CVD Risk Markers and in Fatty Liver Disease

Exenatide has been shown to have beneficial effects on cardiovascular biomarkers. Acute exenatide administration suppressed postprandial excursions of pro-atherogenic lipid and lipoprotein in overweight/obese adults with impaired glucose tolerance or recent onset T2DM [82,83]. Fifty-seven percent of these patients were being treated with statins for dyslipidemia. However, one injection of exenatide was still able to markedly reduce postprandial elevation of triglycerides, apolipoprotein B-48, apolipoprotein-CIII, remnant-like particles-cholesterol and remnant-like particles-triglyceride compared with baseline. In another study, ExBID significantly improved markers of oxidative stress (prostaglandin F2α) and cardiovascular inflammation (high sensitivity C-reactive protein [hsCRP]), independent of baseline HbA_1c_, body weight, and BMI [84]. 

ExBID also had beneficial effects on liver fat content in patients with type 2 diabetes, in whom nonalcoholic fatty liver disease is prevalent [85]. ExBID significantly increased plasma adiponectin, reduced hepatic fat content (from mean 12.1% to 4.7%), and plasma triglyceride concentrations (-38%), despite no significant change in body weight. In addition, the hepatic injury biomarkers aspartate aminotransferase (AST) and alanine aminotransferase (ALT) were significantly decreased by ExBID, suggesting beneficial effects on fatty liver disease.

## EXENATIDE BID EFFICACY IN LATE STAGE CLINICAL TRIALS

### Overview

In controlled trials ranging in treatment duration from 12 to 52 weeks where HbA_1c_ reduction was a primary endpoint, ExBID as monotherapy and in various combinations with MET, SFU, TZD, and insulins reduced mean HbA_1c_ by -0.8% to -1.7% in T2DM patients with mean baseline HbA_1c_ values ranging from 7.5% to 10.2% (Tables **1** and **2**) [86-120].^1^,^2^ The percentage of patients with T2DM achieving HbA_1c_ values ≤7% with ExBID ranged from 20% to 79%, depending upon the baseline characteristics of the patient populations, their concomitant medications, and the active treatment regimen. In a study where weight reduction was the primary endpoint, patients with T2DM with a mean baseline HbA_1c_ of 7.7% still had mean HbA_1c_ reductions of -0.6% more than PBO when ExBID was added to intensive lifestyle modification [121]. 

Meta-analyses of large clinical trials have demonstrated a positive correlation between baseline HbA_1c_ and the magnitude of HbA_1c_ response to therapy, independent of drug class or mechanism of action [122,123]. Consistent with these reports, ExBID-treated patients with a high mean baseline HbA_1c_ of 10.2% had a mean HbA_1c_ change of -1.74% after 24 weeks of treatment [86]. This cohort was insulin-naive, had a mean diabetes duration of 9 years, and had failed to maintain glycemic control during treatment with MET plus SFU. In a study that allowed up-titration of insulin glargine to help achieve glycemic goals in all treatment arms, patients with a more moderate mean baseline HbA_1c_ of 8.3% had a mean HbA_1c_ reduction of -1.74% after 30 weeks of ExBID treatment [90]. Notably, these patients had a longer mean diabetes duration (12 years) than in the Bergenstal *et al.* [86] study and had failed to achieve glycemic control on more aggressive therapy (insulin glargine with or without MET±TZD). A *post hoc* analysis of pooled data from 17 ExBID clinical trials encompassing 2,096 patients found reductions in glycemic parameters and body weight across a wide range of background antidiabetes therapies [124].

Body weight reductions have consistently been associated with ExBID therapy, despite wide variability in T2DM duration and patient characteristics at baseline, concomitant medications, and study designs (Tables **1** and **2**) [86-120].^1^,^2^ Mean weight change with ExBID ranged from -1.2 kg to -8.0 kg in exposure periods ranging from 12 to 52 weeks. The proportion of ExBID-treated patients who had reductions in both HbA_1c_ and body weight was reported in two controlled trials: 66% versus 36% with PBO [99] and 71% versus 54% with PBO [107]. A small percentage of patients may gain weight while receiving exenatide therapy. 

### Pivotal Clinical Trials, Pivotal Trial Extensions, and Monotherapy

**Fig. (7):**
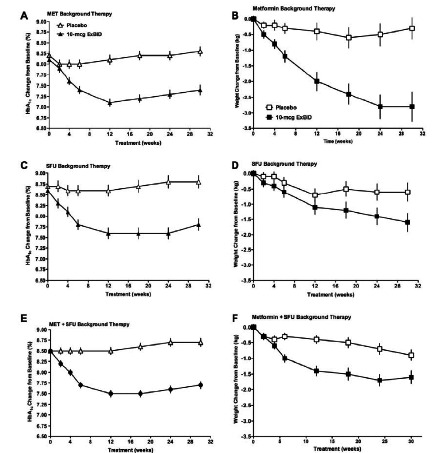
ExBID treatment reduced HbA_1c_ and body weight in patients with T2DM in the three pivotal phase 3 clinical trials on differing oral antidiabetes drug backgrounds. At Week 30, significant HbA_1c_ and weight reductions were observed in all ExBID groups compared with placebo (p<0.05). LS mean±SEM. (Adapted from references [92,95,105]).

The three primary, 30-week, PBO-controlled exenatide pivotal trials were conducted in patients with T2DM not achieving glycemic control with MET and/or SFU [92,95,105]. Treatment with ExBID resulted in mean HbA_1c_ reductions ranging from -0.8 to -0.9% (Fig. **7**). For patients treated with ExBID+SFU, when baseline HbA_1c_ was <9% the mean HbA_1c_ change from baseline was -0.65% and when baseline HbA_1c_ was ≥9% the mean HbA_1c_ change was -1.2% [92]. For patients treated with ExBID+MET+SFU, when baseline HbA_1c_ was (9% the mean HbA_1c_ change from baseline was -0.5% and when baseline HbA_1c_ was ≥9% the mean HbA_1c_ change was .3% [105]. Thus, ExBID reduced HbA_1c_ to a greater extent in patients with higher baseline HbA_1c_, ie, worse glycemic control. The percentage achieving an HbA_1c_≤7% after 30 weeks of ExBID treatment in these pivotal trials ranged from 30% to 46%. These patients also had progressive body weight reductions ranging from -1.6 to -2.8 kg. These improvements in glycemic control and body weight are especially notable, because no specific diet or exercise counseling, nor caloric restriction, were required by the study protocols. 

**Fig. (8):**
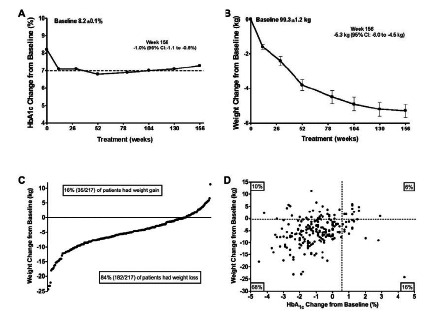
Long-term efficacy of ExBID on HbA_1c_ (**A**) and body weight (**B**). (**C**) Individual patient data showing that the majority of exenatide-treated patients (84%) lost weight after 3-years of ExBID therapy. (**D**) Individual patient data showing that the majority of exenatide-treated patients (68%) both lost weight and had reduced HbA_1c_ after 3-years of ExBID therapy. N=217 (Reprinted with permission from reference [**106**]).

A series of reports followed patients with T2DM originally enrolled in these first three ExBID pivotal trials during open-label extensions with up to 3.5 years of exposure (Tables **1** and **2**, Fig. **8**) [87,93,106,110,111]. The majority of ExBID-treated patients with T2DM who continued therapy maintained HbA_1c_ reductions in combination with progressive weight loss, with 84% of patients reporting weight loss at Week 104 (2-year evaluable population N=521; 54% completed) [94]. After 3 years of ExBID, patients with T2DM on a background of MET, SFU, or MET+SFU had a mean HbA_1c_ reduction of -1.0%, and 46% achieved an HbA_1c_≤7% [106]. The sustained nature of the glycemic control associated with ExBID therapy may, in part, reflect effects on pancreatic β-cell secretory function, as evidenced by sustained improvements in HOMA-B out to 3 years. Fifty percent lost ≥5% of baseline body weight and 68% had reductions in both HbA_1c_ and weight. This is in contrast to the weight gain associated with many other antidiabetic agents as glycemia improves [125]. After 3.5 years of therapy, 10-mcg ExBID therapy significantly improved a number of cardiovascular risk factors: mean change from baseline was -12% for triglycerides, -6% for LDL-C, -5% for total cholesterol, +24% for HDL-C, -2% for systolic BP, and -4% for diastolic BP [106]. The 25% of ExBID-treated patients who lost the most weight had the greatest improvements in triglycerides, HDL-C, and blood pressure. However, for the entire cohort there were minimal correlations with weight change. These data suggest that ExBID may improve overall cardiometabolic risk factors by mechanisms that are partially dependent on weight loss and partially weight-independent. Data also suggest a role for exenatide in improving cardiometabolic and hepatic biomarkers in patients with T2DM. Elevated serum ALT is a biomarker of hepatic injury, often caused by the nonalcoholic fatty liver disease associated with obesity and T2DM. After 3 years of ExBID, mean ALT in patients with elevated baseline ALT declined progressively (-26%) and 41% had ALT normalization. Although the 25% of patients who lost the most weight had the greatest reduction in ALT, weight change in the entire cohort was only mildly correlated with baseline ALT (r= -0.01) or ALT change r=0.31).

The efficacy of ExBID monotherapy in treatment-naive patients with T2DM was explored in two PBO-controlled clinical trials, one was preliminary [126] and one was phase 3 [108]. In the preliminary study, ExBID monotherapy significantly reduced hyperglycemia and body weight over 28 days of treatment. In the phase 3 study, 24 weeks of ExBID monotherapy significantly reduced mean HbA_1c_ by -0.9%, compared with -0.2% in the PBO group. An HbA_1c_ ≤7% was achieved by 46% with ExBID, compared with only 29% with PBO. Mean daily six-point self-monitored blood glucose concentrations were significantly reduced with ExBID monotherapy compared with PBO (daily mean change -27 mg/dL versus -5 mg/dL, respectively). In addition, β-cell function measured by HOMA-B improved by 28% in the ExBID group compared with 6% in the PBO group. 

### Insulin Comparator Studies

ExBID has generally demonstrated non-inferiority to insulin glargine and biphasic insulin aspart [86,88,94,102, 109,127]. In addition, weight loss was associated with ExBID therapy, compared with weight maintenance or weight gain with insulin therapy. In one study, a strategy of aggressive insulin dose titration reduced HbA_1c_ more with insulin than with ExBID, albeit with greater weight gain in the insulin group [86]. 

Recently, the data from four ExBID versus insulin comparator studies [94,102,109,127] were pooled (1423 patients) and analyzed [128]. Treatment ranged from 16 to 52 weeks in duration. Overall glycemic control with ExBID (mean HbA_1c_ change -1.2%) was non-inferior to insulin (mean HbA_1c_ change -1.1%) at 6 months, and patients who continued treatment through 52 weeks showed sustained reductions in HbA_1c_ from baseline (ExBID, -1%; insulin, -0.9%). When stratified by HbA_1c_ change from baseline tertiles, ExBID and insulin were associated with comparable reductions in each tertile, with the greatest mean reductions of -1.7% (ExBID) and -1.7% (insulin) observed in the tertile with the highest baseline HbA_1c_ range (9.0% to 12.7%). ExBID was associated with weight loss (mean -2.0 kg) versus weight gain with insulin (mean +1.8 kg) at Week 26; the corresponding values at Week 52 were -3.1 kg vs +1.9 kg. Overall, more ExBID-treated patients (70%) experienced weight loss than did those treated with insulin (21%). For patients with an elevated systolic BP at baseline (≥130 mmHg), ExBID reduced systolic BP to a significantly greater extent than insulin (mean -8.1 vs -3.6 mmHg). The frequency of nocturnal, mild-to-moderate hypoglycemia was lower with ExBID (15%) than with insulin (29%). 

### Other CVD Effects

**Fig. (9):**
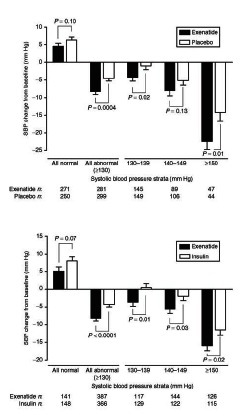
Pooled analysis of systolic blood pressure data from six ExBID clinical trials including 2,171 patients with T2DM. (**A**) Systolic blood pressure change from baseline in patients treated with ExBID or placebo for 6 months. Mean+SEM. (**B**) Systolic blood pressure change from baseline in patients treated with ExBID or insulin for 6 months. Mean+SEM. (Reprinted with permission from reference [**129**]).

ExBID effects on BP were analyzed in more depth by pooling data from patients with T2DM treated in six comparator controlled (PBO or insulin) clinical trials of 24 to 52 weeks duration (Fig. **9**) [129]. All patients continued existing antidiabetes, antihypertensive, and dyslipidemia medications. In the pooled analysis, 6 months of ExBID therapy was associated with a significantly greater reduction in systolic BP than was PBO or insulin. Patients with elevated baseline systolic BP (≥130 mmHg) appeared to drive the BP changes observed in the overall population. This subgroup had mean systolic BP reductions with ExBID of -8.3 mmHg versus -4.5 mmHg with PBO; and -8.3 mmHg with ExBID versus -4.2 mmHg with insulin. Although the majority of exenatide-treated subjects lost weight, weight loss was only weakly correlated with systolic BP change (r=0.09), again suggesting exenatide effects were not solely mediated by weight loss.

Clinical trial data from patients with T2DM treated with ExBID or insulin glargine on a background of MET for one year yielded additional insight into the complex metabolic changes associated with exenatide therapy [130,131]. Exenatide treatment reduced total body fat mass and improved the profile of biomarkers for CVD risk compared with insulin glargine. ExBID also significantly reduced post-meal proatherogenic triglycerides, apoB48, VLDL-C, and free fatty acid excursions, as well as the oxidative stress markers MDA and oxLDL-to-LDL ratio compared with insulin glargine. Body weight (-6%), waist circumference (-5%), and total body fat mass (-11%) were all reduced compared with insulin glargine. In addition, there were significant changes in total adiponectin (+12%) and hsCRP (-61%) compared with insulin glargine. In contrast, insulin glargine only significantly reduced endothelin-1 (-7%) compared with ExBID. Notably, the adiponectin and hsCRP changes in the ExBID arm were independent of the changes in total fat mass (adiponectin: r= -0.224; hsCRP: r= -0.023). One year of ExBID also significantly reduced hsCRP in two other comparator-controlled clinical trials [96,97]. 

Acute exenatide effects on postprandial lipids, remnant lipoproteins, and apolipoproteins in subjects with impaired glucose tolerance or recent onset T2DM were also reported by Schwartz *et al.* [82]. A single exenatide injection prior to a high-fat meal significantly reduced postprandial triglyceride, apoB48, apoCIII, non-esterified fatty acid, and remnant lipoprotein excursions, compared with PBO. Postprandial excursions of total apoB and LDL-C were not changed. These metabolic effects were not affected by the degree of ambient hyperglycemia nor by dyslipidemia treatment with statins. 

## STUDY ON THE FEASIBILITY OF CONTINUOUS EXENATIDE DOSING 

In order to evaluate the feasibility of transitioning from twice daily to once weekly exenatide administration in humans, 12 patients with T2DM treated with diet/exercise or MET received continuous infusions of exenatide or PBO subcutaneously over one-day intervals, delivered via pumps [131]. Exenatide reached therapeutic plasma concentrations (≥50 pg/ml) and significantly reduced plasma glucose during both prandial and fasting states, confirming day-long glycemic control with continuous peptide exposure. There were no serious or severe adverse events, and no cases of hypoglycemia. The most common adverse events with exenatide treatment were mild nausea and/or vomiting. 

## EXENATIDE ONCE WEEKLY (QW)

### Overview

**Fig. (10):**
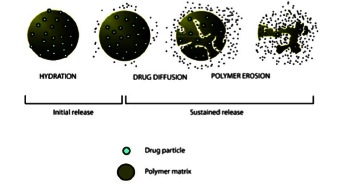
ExQW mechanism of drug release from poly-(D,L-lactide-co-glycolide) microspheres. (Reprinted with permission from reference [**133**]).

The formulation of exenatide into a 2-mg once weekly (ExQW) agent differs from ExBID due to encapsulation of the exenatide peptide within poly-(D,L-lactide-co-glycolide) microspheres (Fig. **10**) [132]. After mechanical suspension and subcutaneous injection, ExQW microspheres hydrate in situ and adhere to each other to form an amalgam. A small amount of exenatide (1-2% of the total area under the plasma concentration-time curve) that is loosely bound to the amalgam surface reaches the circulatory system within the first few hours post-injection. Exenatide located in deeper interstices diffuses out more slowly with a T_max_ of approximately 2 weeks. Fully encapsulated exenatide, that is initially inaccessible to diffusion, releases with a T_max_ of approximately 7 weeks as the microspheres hydrolyze and are eliminated as carbon dioxide and water. Plasma exenatide concentrations reach the therapeutic range by 2 weeks post-injection and achieve steady state by 6 to 7 weeks post-injection. 

In phase 3 clinical trials, 2-mg ExQW significantly reduced mean HbA_1c_ by -1.3% to -1.9% during controlled treatment periods ranging from 24 to 30 weeks [113,114,116-118].^1^ Mean body weight change ranged from -2.3 to -3.7 kg. Further, the proportion of ExQW-treated patients with reductions in both HbA_1c_ and body weight ranged from 70% to 79%.

### Initial Clinical Testing

The initial phase 2 clinical trial of ExQW evaluated effects in patients with T2DM randomized to ExQW or PBO for 15 weeks on a background of MET or diet/exercise [133]. By Week 2, plasma exenatide concentrations exceeded the minimally effective level (approximately 50 pg/ml) shown to reduce fasting plasma glucose concentrations and remained within the target therapeutic range throughout the remainder of the treatment period. ExQW patients had an HbA_1c_ reduction of -1.7±0.3% compared with an HbA_1c_ increase of 0.4±0.3% in the PBO group. In addition, 86% of patients in the ExQW group achieved an HbA_1c_≤7%, compared with none in the PBO group. Body weight was unchanged in the PBO group, but decreased in the ExQW group by 3.5% (-3.8±1.4 kg). Mild nausea was the most frequent exenatide-associated adverse event (27% versus 15% with PBO). Of note, the incidence of injection site reaction was 7% with ExQW versus 0% with PBO.

### Phase 3 Clinical Trials

Phase 3 ExQW clinical development in patients with T2DM is comprised of the DURATION (Diabetes therapy Utilization: Researching changes in HbA_1c_, weight and other factors Through Intervention with exenatide ONce weekly) program [134]. In the series of DURATION clinical trials completed to date, ExQW has been compared with ExBID (DURATION-1, -5), metformin (DURATION-4), sitagliptin (DURATION-2, -4), pioglitazone (DURATION-2, -4), liraglutide (DURATION-6), and titrated insulin glargine (DURATION-3) on background antidiabetes therapies of diet/exercise, MET alone, or combination OAD therapy (MET, SFU, TZD) (Tables **1** and **2**) [86-120].^1^,^2^ In controlled treatment periods of the DURATION trials (ranging from 24 to 30 weeks), ExQW significantly reduced mean HbA_1c_ by -1.3% to -2.0% from mean baseline HbA_1c_ values ranging from 8.3% to 8.6%. Mean body weight change ranged from -2.3 to -3.7 kg with ExQW treatment from mean baseline weight values ranging from 88 to 103 kg.

**Fig. (11):**
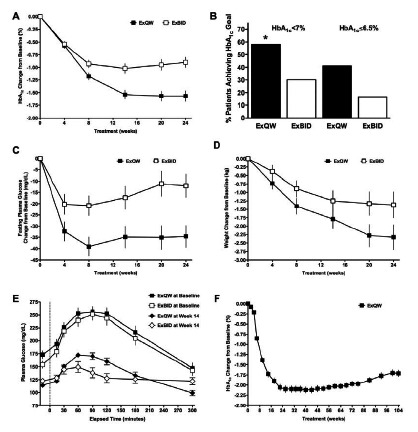
**(A-D) **Comparison of treatment effects for ExQW versus ExBID over 24 weeks in the duration-5 phase 3 clinical trial in treatment-naíve patients with T2DM. (**A**) HbA_1c_ change from baseline. LS mean±SEM. (**B**) Proportion of subjects achieving HbA_1c_ targets at study end. *p<0.0001. (**C**) Fasting plasma glucose change from baseline. LS mean±SEM. (**D**) Body weight change from baseline. LS mean±SEM. ITT population: ExQW n=129; ExBID n=123. (A-D adapted from reference [114]). (**E**) Comparison of postprandial glucose effects after a normal meal in patients treated with ExQW or ExBID at baseline and at Week 14 in the duration-1 phase 3 clinical trial in patients with T2DM. ExQW n=27; ExBID n=24. (Adapted from reference [117]) (**F**) HbA_1c_ change from baseline in the 2-year extension of the duration-1 clinical trial. 2-year completer population: ExQW n=216. LS mean±SEM. (Adapted from reference [119]).

DURATION-1 and -5 directly compared ExQW with ExBID in patients treated with diet/exercise, MET alone, or combination OAD therapy [114,115]. ExQW significantly reduced fasting glucose (Fig. **11**) and glucagon to a greater extent than ExBID. In contrast, ExBID reduced postprandial glucose excursions to a greater extent than did ExQW. During the 30 week controlled portion of DURATION-1, ExQW reduced mean HbA_1c_ by -1.9%. In the open-label extension, patients with T2DM treated with ExQW from baseline had HbA_1c_ reductions of -2.0% at Week 52 [115], -1.7% at Week 104 [119], and -1.6% at Week 156^2^ on a background of diet/exercise, MET, SFU, TZD, or any 2 OADs. Treatment with ExQW was associated with mean weight reductions of -3.7 kg at Week 30 [117], -4.1 kg at Week 52 [115], -2.6 kg at Week 104 [119], and -2.3 kg at Week 156.^2^ In DURATION-5, ExQW reduced mean HbA_1c_ by -1.6% and body weight by -2.3 kg on a background of diet/exercise, MET, SFU, TZD, or any combination of the OADs [114].

The second study with long-term data from an open-label extension (DURATION-2) confirmed that glycemic control could be sustained for up to one year with ExQW [113,120]. At the end of the controlled treatment period treatment of 26 weeks, ExQW on a background of MET reduced mean HbA_1c_ by -1.5%, compared with -1.6% at Week 52. The corresponding weight reductions were -2.3 kg and -1.8±0.5 kg, respectively [114,120].

In the DURATION-6 study, ExQW reduced mean HbA_1c_ by -1.3% after 26 weeks of treatment, compared with -1.5% in the liraglutide treatment arm where the liraglutide cohort underwent a forced titration to the maximum approved dose.^1^ The HbA_1c_ treatment difference was 0.2%, with a 95% confidence interval of 0.08% to 0.34%. Therefore, under the predefined study analysis requiring the upper limit of the HbA_1c_ 95% confidence interval to be <0.25%, ExQW did not meet the primary endpoint of noninferiority to the maximum daily dose of liraglutide. Mean weight reduction was significantly greater in patients treated with liraglutide (-3.6 kg) than with ExQW (-2.7 kg). Both drugs reduced systolic BP, diastolic BP, and a number of cardiovascular risk factors to a similar extent.

In the only study to evaluate ExQW in drug-naíve patients (DURATION-4), mean HbA_1c_ and body weight changes were -1.5% and -2.0 kg, respectively, after 26 weeks of treatment [118]. However, ExQW is not currently recommended for use as first line therapy in patients failing diet/exercise [135].

In the controlled DURATION trials, the proportion of ExQW-treated patients who had reductions in both HbA_1c_ and body weight ranged from 67% to 79% [113,114,116-118].^1^ In DURATION-2 [112], reductions in both HbA_1c_ and body weight occurred in 70% with ExQW, 46% with sitagliptin, and 14% with pioglitazone on a background of MET. In DURATION-3 [116] reductions in both HbA_1c_ and body weight occurred in 79% with ExQW versus 31% with insulin glargine, on a background of MET±SFU. In DURATION-5 [114], reductions in both HbA_1c_ and body weight occurred in 71% with ExQW compared with 51% with ExBID on a background of diet/exercise, MET, SFU, TZD, or a combination of 2 OADs. On the same background therapy, DURATION-1 [117] reported that 73% of ExQW-treated patients had reductions in both HbA_1c_ and body weight, compared with 74% of ExBID-treated patients. In DURATION-4 [118], treatment- naíve patients had reductions in both HbA_1c_ and body weight in 67% with ExQW, 69% with MET, 33% with pioglitazone, and 59% with sitagliptin.

### GLP-1 Receptor Agonism Versus Enhancing Endogenous Incretin Levels

As described in the nonclinical sections of this review, the GLP-1 receptor agonist exenatide is resistant to degradation by the DPP-4 enzyme responsible for rapidly metabolizing endogenous GLP-1. An alternative strategy for targeting the GLP-1 signal transduction pathway has been to inhibit DPP-4 activity, which increases the time that endogenous incretins remain active. Exenatide has been directly compared with the DPP-4 inhibitor sitagliptin in patients with T2DM in several clinical trials. In the DURATION-2 trial, 26 weeks of 2-mg ExQW was compared with 100-mg sitagliptin once daily in patients concomitantly treated with MET [113]. Mean HbA_1c_ reductions were significantly greater with ExQW than with sitagliptin (LS mean change from baseline for ExQW: -1.5%, sitagliptin: -0.9%). In addition, ExQW-treated patients lost a mean of -2.3 kg, compared with mean weight loss of -1.5 kg in sitagliptin-treated patients. When patients were later switched from sitagliptin to ExQW for an additional 26 weeks of treatment, mean HbA_1c _was further reduced by -0.3% and mean weight by -1.1 kg [120]. In the DURATION-4 clinical trial, 26 weeks of 2 mg ExQW was compared with 100-mg sitagliptin once daily in treatment-naíve patients [118]. Mean HbA_1c_ reductions were significantly greater with ExQW than with sitagliptin (LS mean change from baseline for ExQW: -1.5%, sitagliptin: -1.2%). LS mean body weight changes were significantly greater with ExQW (-2.0 kg) than with sitagliptin (-0.8 kg). As described in the clinical pharmacology section above, ExBID treatment was also associated with greater effects on glycemic indicators than was sitagliptin [69,74].

## EXENATIDE ADVERSE EVENTS IN CLINICAL TRIALS AND ROUTINE CLINICAL USE

### Overview

ExBID was approved for human use in 2005 by the U.S. Food and Drug Administration and there is a large clinical trial database of safety events available for analysis [137]. The most common side effects are gastrointestinal in nature, mild, and transient. Nausea is the most prevalent adverse event at drug initiation, and it tends to decrease in frequency with continued exenatide exposure.

**Fig. (12):**
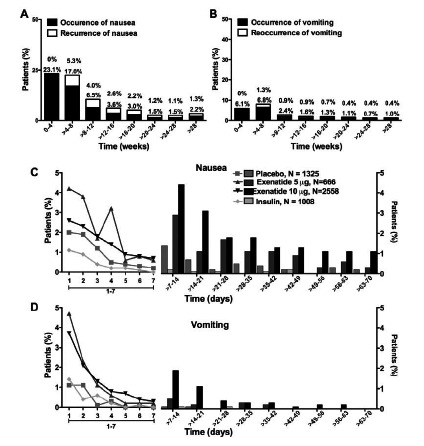
Incidence, recurrence, and duration of nausea and vomiting with ExBID (4-week lead-in period with 5 mcg BID followed by a dose increase to 10 mcg BID for the duration of the trial). ExBID n=2558. Placebo n=1325. Insulin n=1008. (**A**) Occurrence and recurrence of nausea over time (grouped into 4-week intervals). (**B**) Occurrence and recurrence of vomiting over time (N=2558). Each event is attributed to a defined period according to the event onset date, and recurrence of nausea/vomiting is defined as an event with onset during the defined period and previous period. Percentages are based on number of subjects who remained in the trial during the defined period. (**C**) Duration of nausea. (**D**) Duration of vomiting. The duration of the nausea/vomiting event is calculated as the resolution date (or the last participation date if an event is ongoing at the time of study termination) minus the event onset date plus 1. (Reprinted with permission from reference [**137**]).

Recently, MacConell *et al.* [137] published an integrated analysis of 5594 patients with T2DM from 19 comparator-controlled exenatide BID clinical trials.^3^ In this report, representing over 1500 patient-years of exposure, the exenatide BID safety profile was compared with the safety profile of a pooled comparator (PBO, insulin aspart, or insulin glargine) cohort treated for 12 to 52 weeks on background therapies of diet/exercise, MET, SFU, TZD, and OAD combinations. Both cohorts had comparable baseline characteristics. For the 3261 exenatide-treated subjects, 44% were female with a mean age of 56 years and a mean BMI of 32 kg/m^2^. Mean baseline HbA_1c_ was 8.3% and mean treatment exposure was 166 days. For the 2333 subjects in the pooled comparator cohort, 46% were female with a mean age of 56 years and a BMI of 32 kg/m^2^. Mean HbA_1c_ was 8.3% and mean treatment exposure time 171 days. No specific safety signals of concern were identified with exenatide use. Both cohorts had comparable rates of serious adverse events, discontinuations due to serious adverse events, and deaths (exenatide: 3.6%, 0.8%, and <0.1%, respectively; pooled comparator: 3.9%, 0.7%, and <0.1%, respectively). Consistent with the results from individual clinical trials, transient mild-to-moderate nausea was the most frequent side effect associated with exenatide (36.9% versus 8.3% in the pooled comparator) (Fig. **12**). Overall, gastrointestinal adverse events were more frequently observed with exenatide than with comparator treatment (51% versus 21%, respectively). Gastrointestinal adverse events were also the most common reason for discontinuation of clinical trial participation in the exenatide cohort. Vomiting had a composite frequency of 14% compared with 3% in the pooled comparator group, while diarrhea had a composite frequency of 11% versus 5% in the pooled comparator group. However, nausea and vomiting decreased over time in the exenatide group (independent of dose), with a substantial reduction in incidence rate after 8 weeks of treatment. Of the other most-frequent adverse events (≥5% frequency), only dizziness occurred significantly more frequently with exenatide than with comparator exposure (5% versus 3%, respectively).

In another integrated analysis, MacConell *et al.* published a preliminary report of adverse events from 4,328 patients with T2DM treated in 8 randomized phase 3 ExQW trials. The most frequent adverse events with ExQW were gastroin testinal and injection site-related. These adverse events were generally mild and incidences decreased over time. Gastrointestinal adverse events were lower with ExQW than with ExBID (35% vs 46%, respectively) or another GLP-1 receptor agonist, liraglutide (26% vs 42%, respectively). Hypoglycemia occurred infrequently unless there was concomitant SFU use. The adverse events of pancreatitis, pancreas cancer, thyroid cancer, and renal-related problems were generally similar for ExQW, ExBID, and a pooled comparator group of patients treated in these trials with non-GLP-1 receptor agonists.

An overview of selected adverse effects observed in individual phase 3 exenatide clinical trials are summarized in Table **3** [86-20].^1^,^2^ Nausea was the most frequent exenatide side effect, ranging from 12% to 57% upon initiation of 10-mcg exenatide BID (preceded by 4 weeks of 5-mcg ExBID) and 7% to 26% upon first initiation of 2-mg ExQW. Nausea tended to be mild-to-moderate and decreased in frequency with longer duration of exenatide exposure, independent of formulation (BID or QW) [136]. Vomiting had a lower incidence than nausea, ranging from 4% to 22% upon initiation of 10-mcg ExBID and 4% to 11% upon first initiation of 2-mg ExQW. Due to differences in the pharmacokinetic profiles of ExBID and ExQW upon initiation of dosing (rapid achievement of therapeutic concentration of exenatide with ExBID versus 6-7 weeks to achieve final therapeutic concentration of exenatide with ExQW), the lessened incidence and intensity of gastrointestinal side effects with ExQW is not unexpected. 

### Accidental Overdosing

Several reports have described the adverse events associated with exenatide overdosing [138,139]. During one clinical trial, three patients were accidently injected with approximately 100-mcg of exenatide [138]. All three presented with severe nausea and vomiting, and one of them experienced severe hypoglycemia that resolved with intravenous administration of 5% dextrose. All three patients recovered without further sequelae. In another report, Cohen *et al.* [139] discussed the emergency room presentation of a 40-year-old morbidly obese woman with T2DM who self-injected 90-mcg of exenatide in addition to her usual morning medications. The patient presented with dizziness, weakness, nausea, one episode of vomiting, normal vital signs, normal laboratory values, and fasting blood glucose ranging from 84 to 109 mg/dL upon hourly monitoring. Treatment consisted of metoclopramide intravenously. The patient did not become hypoglycemic and fully recovered. In summary, accidental exenatide overdosing was associated with short-term gastrointestinal sequelae and the possibility of an increased hypoglycemia risk. Adverse events due to overdosing have so far been managed successfully with prompt and appropriate medical care.

### Hypoglycemia

In the MacConell *et al.* integrated analysis, the overall incidence of hypoglycemia was comparable between groups without SFU use, but between-group differences became apparent when hypoglycemic events were evaluated based on concomitant use of insulin-secretogogue SFU therapy [136]. The incidence rate of mild-to-moderate or major hypoglycemia without concomitant SFU therapy was 3.1% with exenatide and 2.7% with comparators. In contrast, hypoglycemia was more prevalent when exenatide was administered with a SFU (26.5%) than in the pooled comparator cohort (20.7%). 

In individual phase 3 exenatide clinical trials major/severe hypoglycemia was rare with 10-mcg ExBID or 2-mg ExQW exposure (Table **3**) [86-120]^1^,^2^, ranging from <1% to 4%. The incidence of all cases of reported hypoglycemia ranged from 0% to 58% upon initiation of 10-mcg ExBID (preceded by 4 weeks of 5-mcg exenatide BID) and 1% to 8% upon first initiation of 2 mg ExQW. Concomitant use of insulin-secretogogue SFU was more often associated with hypoglycemic events than was concomitant MET use. Insulin was usually, but not always, associated with more hypoglycemia than exenatide. In addition, nocturnal hypoglycemia was significantly more frequent in subjects treated with insulin glargine or aspart than with exenatide.

In summary, major/severe hypoglycemia has rarely been observed with ExBID or ExQW use. In the absence of concomitant SFU therapy, the risk of mild-to-moderate hypoglycemia is low. Concomitant SFU increases the risk of hypoglycemic events and SFU discontinuation should be considered when initiating exenatide. ExBID use is associated with a reduced incidence of nocturnal hypoglycemia compared with insulin.

### Pancreatitis

**Fig. (13):**
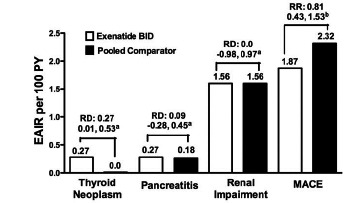
Adverse events of interest. Exposure-adjusted incidence rate (EAIR) and risk difference of thyroid neoplasm, pancreatitis, renal impairment, and major adverse cardiac events (MACE). ExBID n=3261. Pooled comparator n=2333. MACE n=2316 and 1629, respectively. Thyroid neoplasm includes benign neoplasm of thyroid gland and malignant thyroid neoplasm. Pancreatitis includes acute pancreatitis and chronic pancreatitis. Renal impairment includes renal failure. MACE includes stroke, myocardial infarction, cardiac mortality, acute coronary syndrome, and revascularization procedures. ^a^95% confidence interval for the risk difference (RD, ExBID incidence rate [%] minus pooled comparator incidence rate [%]). ^b^MACE analysis: risk ratio (RR) and 95% confidence interval for the RR. PY=patient-years. (Reprinted with permission from reference [137]).

In postmarketing data, exenatide administration has been associated with acute pancreatitis, including fatal and non-fatal hemorrhagic or necrotizing pancreatitis. Of note, acute pancreatitis has been observed in patients treated with exenatide, but it has not been shown that exenatide was causally associated with this adverse event, as will be discussed below. Patient characteristics associated with an increased risk of pancreatitis are overweight/obesity, alcohol use, smoking status, and prior pancreatitis/cholelithiasis/ cholecystectomy [136]. In the MacConell *et al.* [137] integrated analysis discussed above, composite exposure-adjusted incidence rates per 100 patient-years for pancreatitis were not statistically different between the exenatide and pooled comparator groups (0.27 versus 0.18) (Fig. **13**). In addition, occurrence of acute pancreatitis was rare, with a comparable incidence rate of 0.1% in each cohort. In individual phase 3 exenatide clinical trials, pancreatitis was also rare with ExBID or ExQW exposure (Table **3**) [86-120].^1^,^2^

In an analysis using insurance claims from a large U.S. commercial health database, patients with T2DM initiating ExBID were propensity-score-matched with a similar cohort initiating MET or SFU (glyburide) [140]. In this study, exenatide initiators were more likely to be overweight/obese with dyslipidemia and hypertension. Baseline prevalences of gallstone disease (0.8%) and alcohol dependence syndrome (0.1% to 0.2%) were low and comparable across matched-pairs. The estimated absolute risk of hospitalization associated with a primary diagnosis of acute pancreatitis was 0.13% among patients treated with exenatide compared with 0.13% for the matched MET/Gly cohort suggesting that there was no apparent difference in risk. One limitation of this study was that claims for medical services may not meet the strict clinical definition of acute pancreatitis. 

In another retrospective analysis of a health insurance claims database, the risk of acute pancreatitis was significantly higher for adults with diabetes than in normoglycemic adults (Cox proportional hazard ratio=2.1) [141]. The incidence of acute pancreatitis in the cohort with diabetes was 5.6 cases per 1,000 subject years. Unlike the previous report, this analysis included patients with evidence of chronic pancreatic disease and estimated the risk of acute pancreatitis after adjusting for medical conditions and medications associated with an increased risk of acute pancreatitis. Among patients with T2DM, the risk of acute pancreatitis was comparable in the exenatide-treated cohort and the T2DM control cohort (Cox proportional hazard ratio=1.0). 

A more recent evaluation of patients with diabetes (types 1 or 2), and without prior insurance claims for pancreatic disease, found no increased risk of emergency room treatment or hospitalization associated with acute pancreatitis in patients currently taking exenatide compared with a matched cohort on other antidiabetes drugs (propensity score adjusted risk ratio=0.5) [142]. Past exposure to exenatide, defined as more than 62 days after exhaustion of a patient's drug supply, was associated with an increased risk (risk ratio 2.8). However, after adjustment for all chart-derived patient characteristics associated with an increased risk of pancreatitis (overweight/obese, alcohol use, smoking status, and prior pancreatitis/ cholelithiasis/ cholecystectomy) and contemporaneous claim-identified covariates, the past-time estimate was consistent with no increased risk for acute pancreatitis with past exenatide exposure relative to past exposure to other antidiabetes drugs (odds ratio 1.1).

Based on the medical literature (range 1950 to January 2010), FDA website (range 2005 to August 2008), and Amylin Pharmaceuticals database, Anderson and Trujillo [143] published a review of pancreatitis reports associated with ExBID use. The pancreatitis incident rate during clinical development was 1.8 per 1000 subject years for exenatide, compared with 2.6 per 1000 subject years for PBO and 0.9 per 1000 subject years for insulin. Among the FDA-reviewed post-marketing cases of pancreatitis, all 6 patients with hemorrhagic or necrotizing pancreatitis required hospitalization and 2 died. For the 30 other cases of pancreatitis, 19 patients were female (63%) with a median age of 60 years and a time-to-onset of symptoms ranging from 4 to 300 days after exenatide initiation (median 34 days post-exenatide). In 22 cases of pancreatitis the patients improved after exenatide discontinuation and in 21 cases of pancreatitis the patients required hospitalization. Of note, 27 of the 30 patients had at least one other risk factor for acute pancreatitis. 

Elashoff *et al.* [144] used the U.S. Food and Drug Administration (FDA) adverse event reporting system (AERS) database to analyze putative associations between exenatide and cases of pancreatitis, pancreatic cancer, thyroid tumors, and all cancers. As cautioned by the authors, the FDA AERS database depends on spontaneous reporting and is thus, subject to reporting biases. The control antidiabetes drugs were rosiglitazone (TZD), nateglinide, repaglinide, and glipizide (SFU). Back pain, urinary tract infection, chest pain, cough, and syncope were prospectively defined as the control adverse events. By design, no comparable analyses were performed to assess the rates of pancreatitis and cancer associated with the control drugs. The fundamental assumptions in the analysis were no association between the control drugs and control events, or between exenatide and control events. The period covered was 2004 through the third quarter of 2009.Within this framework, the authors found an increased risk of pancreatitis and pancreatic cancer associated with exenatide compared with the control drugs (odds ratio 10.68). When the subset of pancreatitis reports in 2006 and prior (before the first cautionary letter from the FDA in 2007) were analyzed, the odds ratio for exenatide, compared with control drugs, was 2.57 for pancreatitis, 2.95 for pancreatic cancer, 4.73 for thyroid cancer, and 1.08 for all other cancers. Given the potentially serious safety concerns raised by Elashoff *et al.* [144], the European Association for the Study of Diabetes (EASD) evaluated the report in detail [145]. The EASD acknowledged the importance of continual safety monitoring of diabetes therapeutics, but concluded there was no definitive evidence pointing to an increased cancer risk with incretin-based medications. The EASD reiterated the incompleteness and bias associated with spontaneous adverse events reporting systems such as the FDA database. Also, given the analysis period covered by Elashoff *et al.* [144], there was a significant amount of time before exenatide market introduction when only events with control therapy were collected, possibly introducing further bias. In addition, reported events are not evaluated for their adherence to accepted diagnostic standards. EASD stated that the only robust way of measuring comparative risk is within randomized control trials.

In summary, the incidence of acute pancreatitis is rare in patients with T2DM, whether or not they are being treated with exenatide or other diabetes drugs. However, additional monitoring to compile long-term exposure data is warranted, with randomized, controlled clinical trials as the recommended methodology.

All patients treated with exenatide should be monitored for the signs and symptoms of pancreatitis, including persistent severe abdominal pain, possibly radiating to the back, which may or may not be accompanied by vomiting [135,146]. If pancreatitis is suspected, exenatide should be discontinued. If pancreatitis is confirmed, exenatide should not be restarted. Exenatide should not be used in patients with a history of pancreatitis.

### Neoplasias

In both genders of rats, exenatide caused a dose-related and treatment-duration dependent increase in the incidence of thyroid C-cell tumors at clinically relevant exposures compared to controls [136,146]. A significant increase in malignant thyroid C-cell carcinomas was observed in female rats receiving exenatide at 25-times clinical exposure compared to controls and higher incidences were noted in males above controls in all treated groups at ≥2-times clinical exposure. Other GLP-1 receptor agonists have also been shown to induce thyroid C-cell adenomas and carcinomas in male and female mice and rats at clinically relevant exposures [144]. It is unknown whether exenatide will cause thyroid C-cell tumors, including medullary thyroid carcinoma (MTC), in humans, as the human relevance of exenatide-induced rodent thyroid C-cell tumors could not be determined by clinical or nonclinical studies..

As to the topic of 'thyroid cancer' in humans raised by the Elashoff *et al.* analysis [144], the EASD [145] stated that there are different categories of thyroid cancer, with different molecular characteristics and etiologies. The only form possibly mechanistically-related to incretin-based antidiabetic drugs that could potentially be influenced by therapies leading to enhanced stimulation of the GLP-1R is a cascade leading from C-cell hyperplasia to C-cell adenoma to medullary thyroid carcinoma. Medullary thyroid carcinoma is one of the rarer forms of thyroid tumors and it seems counter-intuitive that a change in the incidence in medullary thyroid carcinoma would change the overall incidence of thyroid carcinomas. A similar viewpoint was expressed by Spranger *et al.* [147] for the Drug Commission of the German Medical Association. According to the authors, the time between tumor induction, tumor development, and metastasis/clinical diagnosis generally extends more than 10 years. By contrast, spontaneous adverse drug reactions in the German national database consistently found the time between initial exenatide exposure and tumor detection to be much shorter (2 to 33 months). Thus, the authors concluded that a role for exenatide in tumor induction was doubtful.

In the MacConell *et al.* integrated analysis [137], a small difference was observed between the exenatide BID and pooled comparator groups in the incidence of thyroid tumors (0.3% versus 0.0%, respectively).

In summary, a causal relationship between the development of neoplasias and treatment with exenatide or other diabetes drugs has not been proven in patients with T2DM. However, additional monitoring to compile long-term exposure data is warranted, with randomized, controlled clinical trials as the recommended methodology. Patients with a personal or family history of medullary thyroid carcinoma or multiple endocrine neoplasia syndrome type 2 should not be treated with exenatide [136,146]. 

### Exenatide Use in Renal Disease

Exenatide is primarily metabolized and cleared in the kidney [148], and exenatide pharmacokinetics are significantly altered (i.e., reduced clearance rate, increased half-life) in nondiabetic adults with end-stage renal disease [149]. In addition, ExBID may induce nausea and vomiting, resulting in transient hypovolemia possibly leading to worsened renal function. Therefore, it is prudent to proceed conservatively in patients with moderate renal impairment (creatinine clearance 30-50 mL/min) and to avoid exenatide use completely in patients with end-stage renal disease or severe renal impairment (creatinine clearance <30 mL/min) [136,146].

In the MacConell *et al.* [137] integrated ExBID analysis, there was no between-cohort difference in exposure-adjusted incidence rates per 100 patient-years for renal impairment/failure (1.6 for each cohort). 

In summary, caution should be used when initiating or escalating exenatide therapy in patients with T2DM and moderate renal impairment. Exenatide should not be used in patients with end-stage renal disease or severe renal impairment (creatinine clearance <30 ml/min), and should be used with caution in patients with renal transplantation.

### Cardiovascular Effects

**Fig. (14):**
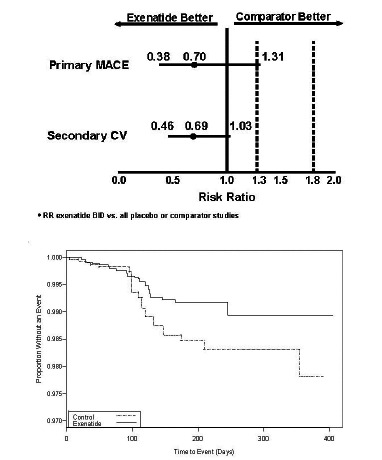
(**A**) Relative risk of primary MACE and secondary cardiovascular endpoints with ExBID relative to pooled comparators. (**B**) Weighted Kaplan-Meier plot for patients without a primary MACE event by treatment in controlled ExBID studies. (Reprinted with permission from reference [150].

Ratner *et al.* [150] reported a retrospective analysis of major adverse cardiac events (MACE) in 3945 subjects from 12 randomized exenatide BID clinical trials; 8 PBO-controlled and 4 active comparator-controlled (Fig. **14**). For this evaluation, MACE included cardiovascular mortality, stroke, myocardial infarction, acute coronary syndrome, and revascularization procedures. Of note, in the clinical trials included in this analysis, patients were excluded from study participation if they had a significant history of cardiac disease at screening, or active cardiac disease within 1 year prior to screening. In this population, MACE occurred infrequently. At baseline, mean HbA_1c_ was 8.3% to 8.4%, mean BMI was 31.3 to 31.5 kg/m^2^, mean systolic BP was 131 to 132 mmHg, and mean diastolic BP was 79 mmHg. There were 4 fatal CV events, 2 in each treatment cohort. The exenatide cohort had an exposure-adjusted incidence rate per 100 patient-years of 1.9, versus 2.3 for the comparator cohort. In a weighted Kaplan-Meier plot a significantly higher percentage of the exenatide cohort than the pooled comparator cohort were free of a primary MACE event over 1 year.

The effects of 10-mcg ExBID on heart rate was prospectively evaluated in a small, 12-week, PBO-controlled pilot study [101]. Overweight patients with T2DM with stage-1 hypertension and treated with MET, TZD, or MET+TZD for their hyperglycemia had no change from baseline in heart rate in either treatment arm. Exenatide was not associated with a significant change from baseline in mean 24-hour, daytime, or nighttime heart rate within or between groups.

Overall, available clinical trial data indicate that treatment with exenatide BID does not increase cardiovascular risk in patients with T2DM.

### Anti-exenatide Antibody Formation and Injection-site Reactions

Xenogenic peptide therapies, such as exenatide, often stimulate an antibody response and the clinical impact of this immune response can range from none to life threatening [151]. Antibody rates for all xenogenic peptide therapies can vary from less than 1% to over 70%. However, not all antibodies are neutralizing, nor do all antibody responses result in attenuation of drug efficacy, altered pharmacokinetics/pharmacodynamics, or changes in drug clearance. In the majority of cases, the antibody response to xenogenic peptides is benign, with minimal clinical impact. 

In an integrated analysis of patients with T2DM exposed to ExBID in controlled clinical trials, 35% of all patients were antibody-positive at endpoint (12 to 52 weeks of treatment); 33% of all patients had low antibody titers (≤125) [152]. For patients exposed to ExQW in clinical trials, 57% were antibody-positive at the endpoints of the controlled periods (24 to 30 weeks); but most (88%) had low antibody titers. In ExQW patients, 22% with detectable antibodies had potentially immune-related treatment-related adverse events, versus 12% in antibody-negative subjects and 10% in the pooled comparator control group. Injection-site erythema or pruritus was reported in 13.6% of antibody-positive patients treated with ExQW, compared with 3.1% of antibody-negative patients and 2.9% of comparator control patients. No anaphylactic reactions occurred with either exenatide formulation.

In summary, anti-exenatide antibody responses are common in patients treated with ExBID or ExQW. However, the incidence of all immune-related adverse events is low. Injection-site erythema or pruritus was observed in patients treated with ExQW, but no anaphylactic reactions have occurred with either exenatide formulation.

### Special Populations

One small PBO-controlled study directly compared exenatide safety in elderly patients with T2DM ≥75 years of age (n=15) with middle-aged patients with T2DM ≥45 to ≤65 years of age (n=15) [153]. At baseline, the elderly cohort had mild or moderate renal impairment, while the middle-aged cohort had normal renal function. Treatment was administered over three consecutive days. Nausea and vomiting with ExBID were more prevalent in the elderly cohort (nausea: 6 patients versus control 3 patients; vomiting: 3 patients versus 1 control patient). All other adverse events were similar in both groups, and none were severe in intensity. There were no reports of hypoglycemia.

## FUTURE DIRECTIONS

A once monthly formulation of exenatide (ExQM) is currently undergoing clinical evaluation.^4^ This formulation uses the extended-release microspheres of ExQW with a triglyceride-based diluent to enable delivery of higher exenatide doses less frequently. A 20-week, randomized, open-label, controlled trial was conducted in 121 patients with T2DM treated with diet/exercise, MET, TZD (pioglitazone) or MET+TZD. Three ExQM doses (5, 8, or 11 mg) administered 5 times as directed were compared with ExQW administered 20 times. Sustained plasma exenatide concentrations were achieved with all ExQM doses, although greater peak-to-trough variability in exenatide pharmacokinetic profiles was observed with ExQM than with ExQW. However, the mean trough concentrations remained within the therapeutic range with all ExQM doses. The 8- and 11-mg ExQM doses achieved plasma exenatide concentrations similar to those of ExQW. All three ExQM doses reduced mean HbA_1c_ by -1.3% to -1.5%, compared with an ExQW mean HbA_1c_ reduction of -1.5%. Seventy percent of patients achieved an HbA_1c_(7% on the 11-mg ExQM dose versus 48% with ExQW. Patients dosed with 11-mg ExQM lost -1.1 kg of body weight, compared with -1.4 kg in the ExQW group. The overall safety profile was comparable between ExQM and ExQW. No hypoglycemia was observed. Based on these preliminary results, more extensive clinical trials are planned.

An ongoing, prospective morbidity and mortality outcomes study (EXSCEL) is expected to further elucidate the potential cardiovascular implications of ExQW in patients with T2DM^5^. The primary outcome measure is the time to first cardiovascular event in a combined endpoint consisting of cardiovascular-related death, nonfatal myocardial infarction, or nonfatal stroke. Secondary endpoints include the time to all-cause mortality, time to first cardiovascular event, time to hospitalization for acute coronary syndrome, and time to hospitalization for heart failure. Recruitment for this trial is ongoing.

## SUMMARY AND CONCLUSION

This review presents an overview of the evolution of exenatide as a treatment for T2DM beginning with the realization of the importance of the 'incretin effect', through seminal preclinical discoveries, clinical pharmacology investigations, and phase 3 clinical trials. Currently, a twice daily exenatide formulation is approved for use in patients with T2DM in the U.S., E.U., Japan, and other countries. More recently, a once weekly exenatide formulation was approved for use in the U.S. and EU. In addition, a once-monthly exenatide formulation is undergoing clinical testing. In patients with T2DM, glycemic control is not always achieved, and some therapeutic interventions cause hypoglycemia and weight gain, or possibly exacerbate the risk of CVD. In clinical trials, exenatide treatment has addressed these unmet medical needs with T2DM patients experiencing improved glycemia, weight loss, and a low frequency of hypoglycemia in the absence of concurrent SFU (insulin secretogogue) use. The effects of exenatide have been shown to be mediated through enhanced glucose-dependent insulin secretion, suppressed inappropriately elevated glucagon secretion, slowed gastric emptying, and enhanced satiety. 

In controlled phase 3 clinical trials ranging from 12 to 52 weeks in duration, 10-mcg ExBID reduced mean HbA_1c_ by -0.8% to -1.7%, accompanied by mean weight loss ranging from -1.2 kg to -8.0 kg, plus improvements in a broad range of cardiometabolic risk factors. Efficacy has been demonstrated as monotherapy and in various combinations with metformin (MET), sulfonylureas (SFU), thiazolidinediones (TZD), and insulins. During phase 3 clinical evaluation of a once weekly exenatide (ExQW) formulation in patients with T2DM in controlled trials ranging from 24 to 30 weeks, 2 mg ExQW reduced mean HbA_1c_ by -1.3% to -1.9%, accompanied by mean body weight reductions ranging from -2.3 to -3.7 kg; with 70% to 79% having reductions in both HbA_1c_ and weight. In the DURATION-1 study extension, ExQW was associated with mean HbA_1c_ reductions of -2.0% at Year 1 and -1.7% at Year 2, on a background of diet/exercise, MET, SFU, TZD, or 2 oral antidiabetes drugs; the corresponding mean weight reductions were -4.1 kg and -2.6 kg. 

The most common side effects are gastrointestinal in nature, mild, and transient. Nausea is the most prevalent adverse event and tends to decrease in frequency with continued exenatide exposure. The incidence of hypoglycemia without concomitant SFU therapy is generally low; hypoglycemia incidence increases when exenatide is used in conjunction with a SFU. Rare cases of pancreatitis have been reported, typically at the same incidence rate as for other diabetes drugs. A causal relationship between the development of neoplasias and treatment with exenatide or other diabetes drugs has not been proven in patients with T2DM. However, additional monitoring to compile long-term exposure data is warranted, with randomized, controlled clinical trials as the recommended methodology. Patients with a personal or family history of medullary thyroid carcinoma or multiple endocrine neoplasia syndrome type 2 should not be treated with exenatide. No detrimental cardiovascular effects have been identified. Most patients develop low titer anti-exenatice antibodies with little or no physiological consequences. No anaphylaxis has been reported with exenatide use. Thus, by building upon early observations of anti-hyperglycemic actions of GLP-1 and Gila monster exendin-4, exenatide (synthetic exendin-4) has been successfully developed into a first-in-class diabetes therapeutic.

## Figures and Tables

**Table 1.** Overview of Exenatide Phase 3 Clinical Trial Designs.

Study Citation	Study Dur (wk)	Double Blind?	Multi-National? (#countries)	Analysis Pop	Mean Calc	NCT#^a^
**Exenatide BID**						
Bergenstal 2009 [86]	24	No	No (US)	ITT & per protocol	Mean	00097877
Blonde 2006 [87]	82	No	No (US)	ITT & completers	Mean	00111540
Bunck 2009 [88]	52	No	Yes (3)	ITT & evaluable	Mean	00097500
Bunck 2011 [89]	168	No	Yes (3)	ITT & evaluable	Mean	00097500
Buse 2011 [90]	30	Yes	Yes (5)	ITT	LS Mean	00765817
Buse 2009 [91]	26	No	Yes (15)	ITT & per protocol	LS Mean	00518882
Buse 2004 [92]	30	Yes	No (US)	ITT & evaluable	Mean	00039026
Buse 2007 [93]	104	No	No (US)	ITT & completers	Mean	00111540
Davies 2009 [94]	26	No	No (UK)	ITT	LS Mean	00360334
DeFronzo 2005 [95]	30	Yes	No (US)	ITT & evaluable	Mean	00039013
Derosa 2010 [96]	52	No	No (Italy)	ITT	Mean	NA
Derosa 2011 [97]	52	No	No (Italy)	ITT	Mean	NA
Forti 2008 [98]	12	No	Yes (2)	Per protocol	LS Mean	00359879
Gallwitz 2011 [99]	26	No	No (Germany)	ITT	LS Mean	00434954
Gao 2009 [100]	16	Yes	Yes (4)	ITT & per protocol	LS Mean	00324363
Gill 2010 [101]	12	Yes	Yes (2)	ITT & per protocol	LS Mean	00516074
Heine 2005 [102]	26	No	Yes (13)	ITT & per protocol	LS Mean	00082381
Kadowaki 2009 [103]	12	Yes	No (Japan)	ITT	Mean	00382239
Kadowaki 2011 [104]	24	Yes	No (Japan)	ITT	LS Mean	00577824
Kendall 2005 [105]	30	Yes	No (US)	ITT & evaluable	Mean	00035984
Klonoff 2008 [106]	156 / 182	No	No (US)	ITT & completers	Mean	00111540
Liukus 2010 [107]	26	Yes	Yes (5)	ITT	LS Mean	00603239
Moretto 2008 [108]	24	Yes	Yes (4)	ITT	LS Mean	00381342
Nauck 2007 [109]	52	No	Yes (13)	ITT & per protocol	Mean	00082407
Ratner 2006 [110]	82	No	No (US)	ITT & completers	Mean	00111540
Riddle 2006 [111]	82	No	No (US)	ITT & completers	Mean	00111540
Zinman 2007 [112]	16	Yes	Yes (3)	ITT & per protocol	LS Mean	00099320
**Exenatide QW**						
Bergenstal 2010 [113]	26	Yes	Yes (3)	ITT & evaluable	LS Mean	00637273
Blevins 2011 [114]	24	No	No (US)	ITT & evaluable	LS Mean	00877890
Buse 2010 [115]	52	No	Yes (2)	ITT & evaluable	LS & Geo Mean	00308139
Buse 2011**^1^**	26	No	Yes (21)	ITT	LS Mean	01029886
Diamant 2010 [116]	26	No	Yes (15)	ITT	LS Mean	00641056
Drucker 2008 [117]	30	No	Yes (2)	ITT & evaluable	LS & Geo Mean	00308139
MacConell 2011**^2^**	156	No	Yes (2)	Completers	LS & Geo Mean	00308139
Russell-Jones 2012 [118]	26	Yes	Yes (22)	ITT	LS Mean	00676338
Taylor 2011 [119]	104	No	Yes (2)	ITT & Completers	LS Mean	00308139
Wysham 2011 [120]	52	No	Yes (3)	Evaluable	LS Mean	00637273

a Additional information on these trials is available at clinicaltrials.gov Abbreviations: BID, twice daily; Dur, duration; Geo, geometric; ITT, intent-to-treat; LS, least squares; NA, not applicable; NCT, national clinical trial database; NI, non-inferiority; Pop, population; QW, once weekly; **Δ**Rx, change or different diabetes medication prior to randomization; S, superiority.

**Table 2.** Overview of Exenatide Phase 3 Clinical Studies.^a^

Study Citation	Ther Studied	BG Ther	DO (%)	ITT N	F (%)	White (%)	T2DM Dur (y) ±SD	BL HbA_1c_ (%) ±SD	ΔHbA_1c_ (%) ±SE	Achieved HbA_1c_ ≤7% or <7% (%)	BL BMI (kg/m^2^) ±SD	BL Wt (kg) ±SD	ΔWt (kg) ±SE
**Exenatide BID**													
Bergenstal 2009 [86]	ExBID	MET + SFU	29.8	124	51.6	63.7	9±6	10.2±1.5	-1.75	20	34±7	96.6±24	-1.9
	Aspart QD		16.1	124	51.6	67.7	8±6	10.1±1.8	-2.34	26	34±7	96.9±25	2.8
	Aspart BID		19.4	124	52.4	59.7	10±6	10.3±1.9	-2.76	37	34±7	93.8±24	4.1
Blonde 2006 [87]	ExBID	MET +/or SFU	43.0	551	39	74	7±6	8.4±1.0	-1.1±0.1	48	34±6	98±20	-4.4±0.3
Bunck 2009 [88]	ExBID	MET	16.7	36	36.1	100	6±1	7.6±0.1	-0.8±0.1	NG	31±1	90.6±2.1	-3.6 ±0.6*
	Glarg		9.1	33	33.3	97	4±1	7.4±0.1	-0.7±0.2	NG	30±1	92.4±2.4	1.0±0.8
Bunck 2011 [89]	ExBID	MET	44.4	36	36.1	100	6±1	7.6±0.1	-1.0	NG	31±1	90.6±2.1	-5.7 ±1.3*
	Glarg		48.5	33	33.3	97	4±1	7.4±0.1	-0.5	NG	30±1	92.4±2.4	2.1±1.3
Buse 2011 [90]	ExBID	Glarg ±MET and/or TZD	19	137	49	75	12±7	8.32± 0.85	-1.74 ±0.1*	60*	34±6	95.4±20.4	-1.8 ±0.3*
	PBO		18	122	36	80	12±7	8.50± 0.96	-1.04 ±0.1	35	33±6	93.4±21.2	1.0±0.3
Buse 2009 [91]	ExBID	MET ± SFU	19.5	231	45	91	8±6	8.1±1.0	-0.8±0.1	43	33±6	93.0±19.5	-2.9±0.3
	LIRA		14.2	233	51	93	9±6	8.2±1.0	-1.1±0.1	54	33±6	93.1±20.1	-3.2±0.3
Buse 2004 [92]	ExBID	SFU	29.5	129	42.6	59.7	7±7	8.6±1.2	-0.9 ±0.1*	34.2*	33±6	95±18	-1.6 ±0.3*
	5 mcg ExBID		24.0	125	40.8	61.6	6±5	8.5±1.1	-0.5 ±0.1*	26.7*	303±6	95±22	-0.9±0.3
	PBO		39.8	123	37.4	66.7	6±5	8.7±1.2	0.1±0.1	7.7	34±5	99±18	-0.6±0.3
Buse 2007 [93]	ExBID	MET +/or SFU	45.7	521	41	74	8±6	8.4±1.1	-1.1±0.1	50	34±6	99±20	-4.7±0.3
Davies 2009 [94]	ExBID	MET +/or SFU +/or TZD	16.1	118	29.7	NG	9±5	8.7±0.7	-1.25± 0.09	NG	35±6	101.4±19.8	-2.7 ±0.3*
	Glarg		10.3	117	33.6	NG	8±4	8.5±0.7	-1.26 ±0.09	NG	34±5	97.6±16.4	3.0±0.3
DeFronzo 2005 [95]	ExBID	MET	17.7	113	39.8	79.6	5±5	8.2±1.0	-0.8 ±0.1*	46*	34±6	101±20	-2.8 ±0.5*
	5 mcg ExBID		18.2	110	48.2	77.3	6±6	8.3±1.1	-0.4 ±0.1*	32*	34±6	100±22	-1.6 ±0.4*
	PBO		21.2	113	40.7	72.6	7±6	8.2±1.0	0.1±0.1	13	34±6	100±19	-0.3±0.3
Derosa 2010 [96]	ExBID	MET	6.3	63	52.4	100	NG	8.8±0.7	-1.5	NG	29±2	82.0±8.3	-8.0
	Gli		12.3	65	49.2	100	NG	8.9±0.8	-1.8	NG	29±1	82.4±9.1	+4.3
Derosa 2011 [97]	ExBID	MET	8.8	57	50.9	100	NG	8.7±0.7	-1.2	NG	28±1	80.2±7.5	-5.1
	Gli		9.3	54	51.9	100	NG	8.8±0.8	-1.4	NG	29±1	81.4±8.1	-0.9
Forti 2008 [98]	ExBID at B, D	MET ± SFU ± TZD	11.1	190	58.4	41.0	9±6	8.4±0.9	-1.2±0.1	45.1	31±4	82.7±16.1	-1.3±0.2
	ExBID at L, D		16.0	187	51.3	45.0	8±6	8.5±1.0	-1.1±0.1	32.6	31±4	81.8±14.2	-1.1±0.2
Gallwitz 2011 [99]	ExBID	MET	25.4	181	NG	NG	5±4	7.9±0.8	-1.00	49.2	33±4	NG	-4.1 ±0.2*
	Aspart		20.8	173	NG	NG	5±5	7.9±0.9	-1.14	56.6	33±4	NG	1.0± 0.2
Gao 2009 [100]	ExBID	MET ± SFU	17.5	234	52	0	8±6	8.3±1.0	-1.2*	48*	26±3	69.6±11.2	-1.2*
	PBO		10.3	232	59	0	8±5	8.3±1.0	-0.4	17	26±3	67.9±11.0	-0.1
Gill 2010 [101]	ExBID	MET +/or TZD	21.4	28	32	86	7±4	7.5±0.9	NG	NA	30±3	91.6±15.2	-1.8 ±0.4*
	PBO		11.5	26	58	96	6±4	7.1±0.7	NG	NA	30±4	85.9±12.2	-0.3±0.4
Heine 2005 [102]	ExBID	MET + SFU	19.1	282	45.0	79.8	10±6	8.2±1.0	-1.1	46	31±4	87.5±16.9	-2.3*
	Glarg		9.4	267	43.4	80.5	9±6	8.3±1.0	-1.1	48	31±5	88.3±17.9	+1.8
Kadowaki 2009 [103]	ExBID	SFU ±MET or ±TZD	16.2	38	37.8	0	10± 6	7.9±0.9	-1.4 ±0.1*	79.4*	26±5	70.3±15.9	-1.3±0.3
	5.0 mcg ExBID		10.8	37	32.4	0	11± 6	7.9±0.8	-1.2 ±0.1*	71.4*	25±3	65.6±9.8	-0.2±0.3
	2.5 mcg ExBID		8.1	38	29.7	0	15±11	8.0±0.8	-0.9 ±0.1*	50.0*	24±3	64.9±11.6	-0.08±0.2
	PBO		2.5	40	25.0	0	12±6	8.1±0.7	0.02± 0.1	5.1	26±5	71.1±14.0	-0.7±0.2
Kadowaki 2011 [104]	ExBID	SFU±MET or SFU±TZD	27.4	72	31.9	0	12±7	8.2±1.0	-1.6* ±0.1	71.0*	26±4	69.1±11.2	-1.5 ±0.3*
	5 mcg ExBID		9.7	72	31.9	0	12±6	8.3±0.8	-1.3 ±0.1*	67.1*	25±4	67.0±11.5	-0.4±0.3
	PBO		5.7	35	31.4	0	12±7	8.1±0.9	-0.3±0.2	15.2	26±4	70.3±13.3	-0.5±0.4
Kendall 2005 [105]	ExBID	MET + SFU	17.8	241	40.7	66.4	9±6	8.5±1.1	-0.8 ±0.1*	30*	34±6	98±21	-1.6 ±0.2*
	5 mcg ExBID		15.9	245	40.8	69.0	9±6	8.5±1.0	-0.6 ±0.1*	24*	33±6	97±19	-1.6 ±0.2*
	PBO		23.9	247	44.1	68.4	9±6	8.5±1.0	0.2±0.1	7	34±5	99±19	-0.9±0.2
Klonoff 2008 [106]	ExBID	MET, + SFU	58.8	527	36	83	8±6	8.2±1.0	-1.0±0.1	46	34±5	99±18	-5.3±0.4
Liukus 2010 [107]	ExBID	TZD ± MET	14	111	40	57	6±4	8.2±0.9	-0.8 ±0.2*	49	34±6	94.5±17.8	-1.4±0.6
	PBO		7	54	43	61	6±5	8.3±0.9	-0.1±0.2	36	33±5	92.6±18.0	-0.8±0.7
Moretto 2008 [108]	ExBID	D/E	14	78	38	72	2±3	7.8±1.0	-0.9 ±0.1*	46*	31±5	86±16	-3.1 ±0.3*
	5 mcg ExBID		13	77	48	65	2±3	7.9±1.0	-0.7 ±0.1*	48*	32±5	85±15	-2.8 ±0.3*
	PBO		12	77	45	66	1±2	7.8±1.0	-0.2±0.1	29	32±5	86±16	-1.4±0.3
Nauck 2007 [109]	ExBID	MET + SFU	21.3	253	47	NG	10±6	8.6±1.0	-1.0±0.1	32*	31±4	85.5±15.7	-2.5 ±0.2*
	Aspart		10.1	248	51	NG	10± 6	8.6±1.1	-0.9±0.1	24	30±4	83.4±15.6	2.9±0.2
Ratner 2006 [110]	ExBID	MET	39	150	31	86	5±5	8.1±1.0	-1.3±0.1	59	34±6	102±21	-5.3±0.8
Riddle 2006 [111]	ExBID	SFU ± MET	45	401	39	75	8±6	8.4±1.0	-1.0±0.1	44	34±6	99±21	-4.0±0.3
Zinman 2007 [112]	ExBID	TZD ± MET	28.9	121	46.3	85.1	7±5	7.9±0.9	-0.9 ±0.1*	62*	34±5	97.5±18.8	-1.8 ±0.3*
	PBO		14.3	112	42.9	82.1	8±6	7.9±0.8	0.1±0.1	16	34±5	96.9±19.0	-0.2±0.3
**Exenatide QW**													
Bergenstal 2010 [113] DURATION-2	ExQW	MET	20.6	160	44	33	6±5	8.6±1.2	-1.5*	~58*	32±5	89±20	-2.3*
	100 mg SITA		13.3	166	48	30	5±4	8.5±1.2	-0.9	~30	32±5	87±20	-0.8
	45 mg PIO		20.6	165	52	39	6±5	8.5±1.1	-1.2	~42	32±6	88±20	2.8
Blevins 2011 [114] DURATION-5	ExQW	D/E, MET, SFU, TZD or any comb	15.5	129	40	63	7±5	8.5±1.1	-1.6 ±0.1*	58.1*	34±6	97.0±20.7	-2.3±0.4
	ExBID		22.8	123	45	55	7±5	8.4±1.2	-0.9±0.1	30.1	33±5	94.3±18.9	-1.4±0.4
Buse 2010 [115] DURATION-1	ExQW	D/E, MET, SFU, TZD or 2 OAD	19	148	43	83	7±6	8.3±1.0	-2.0	71	35±5	103±19	-4.1
	ExBID then ExQW		18	147	47	74	6±5	8.2±0.9	-2.0	71	35±5	102±20	-4.5
Buse 2011^1^	ExQW	MET, SFU, TZD, or any comb	13.2	461	44.9	83.3	8±6	8.4±1.0	-1.3	52.3	32±6	90.9±19.5	-2.7
	LIRA		13.1	450	45.6	81.8	9±7	8.4±1.0	-1.5	60.2	32±5	91.1±19.1	-3.6
Diamant 2010 [116]	ExQW	MET ± SFU	10.3	233	48	82	8±6	8.3±1.1	-1.5 ±0.1*	60*	32±5	91.2±18.6	-2.6 ±0.2*
	Glarg		6.3	223	45	85	8±6	8.3±1.0	-1.3±0.1	48	32±5	90.6±16.4	1.4±0.2
Drucker 2008 [117]	ExQW	D/E, MET, SFU, TZD or 2 OAD	13.5	148	45.0	83	7±6	8.3±1.0	-1.9 ±0.1*	77*	35±5	102±19	-3.7±0.5
	ExBID		11.6	147	49.0	73	6±5	8.3±1.0	-1.5±0.1	61	35±5	102±21	-3.6±0.5
MacConell 2011^2^	ExQW	D/E, MET, SFU, TZD or 2 OAD	34	295	NG	NG	7±5	8.2±1.0	-1.6	57	NG	101±18	-2.3
Russell-Jones 2012 [118]	ExQW	D/E	15.3	248	44	68.1	3±3	8.5±1.2	-1.5±0.1	63	31±5	88±19	-2.0±0.2
	MET		13.4	246	37	65.0	3±4	8.6±1.2	-1.5±0.1	55	31±6	86±20	-2.0±0.2
	PIO		18.4	163	41	67.5	3±4	8.5±1.2	-1.6±0.1	61	31±5	86±18	1.5±0.3
	SITA		14.1	163	42	69.3	3±4	8.5±1.3	-1.2±0.1	43	32±5	89±19	-0.8±0.3
Taylor 2011 [119]	ExQW	D/E, MET, SFU, TZD or 2 OAD	27	295	47	79	7±5	8.2±1.0	-1.7±0.1	60	35±5	101±19	-2.6±0.5
Wysham 2011 [120]	ExQW	MET	35.6	160	43	32	6±5	8.6±1.2	-1.6±0.1	58	32±5	90±19	-1.8±0.5
	SITA then ExQW		21.7	166	42	35	6±5	8.5±1.1	-1.8±0.5	53	32±5	88±21	-1.1±0.3
	PIO then EQW		39.4	165	49	39	6±5	8.4±1.0	-1.6±0.1	59	32±5	86±21	-3.0±0.3

Note: when 'ExBID' is listed without dose, dose was 10 mcg BID. When ExQW listed without dose, dose was 2 mg.

* p<0.05 improvement in HbA_1c_ or weight versus control group.

a Portions of table adapted from: Aroda V, Henry R, Han J, *et al.* A meta-analysis and systematic review of the efficacy of GLP-1 receptor agonists and DPP-4 inhibitors. Clin Ther. 2012;34:1247-58.e22

**Table 3.** Overview of Nausea, Vomiting, Hypoglycemia, and Selected Treatment-Emergent Adverse Events from Phase 3 Exenatide Clinical Trials

Citation	Treatment	Nausea (%)	Vomiting (%)	Hypo (%)	Major/ Severe Hypo?	Other Relevant Observations
**EXENATIDE BID CLINICAL TRIALS**						
Bergenstal 2009 [86]	ExBID+MET+SFU	29	NG	29	No	1.28 hypo/subj-y.
	Aspart QD+MET+SFU	9	NG	56	3.2%	4.02 hypo/subj-y. P=0.0013 vs Ex
	Aspart BID+MET+SFU	8	NG	61	4.8%	5.25 hypo/subj-y. P<0.0001 vs Ex
Blonde 2006 [87]	ExBID+MET +/or SFU	14-29	NG	7-12	0.7%	The severe hypo event occurred in a patient treated with SFU
Bunck 2009 [88]	ExBID+MET	50	NG	8.3	No	1 pancreatitis, resolved after Ex stopped
	Glarg+MET	NG	NG	24.2	No	
Bunck 2011 [89]	ExBID+MET	38.1	9.5	19.0	No	
	Glarg+MET	NG	8.0	28.0	No	
Buse 2011 [90]	ExBID+Glarg	41	18	25.0	No	1.4 hypo/subj-y Nocturnal: 17% hypo
	PBO+Glarg	8	4	29.0	1%	1.2 hypo/subj-y. P=0.49 vs Ex Nocturnal: 26% hypo
Buse 2009 [91]	ExBID+MET±SFU	28.0	9.9	34.5	0.9%	2.6 minor hypo/subj-y. Major hypo had SFU
	LIRA+MET±SFU	25.5	6.0	26.0	No	1.9 minor hypo/subj-y. P=0.0131 vs Ex 1 mild pancreatitis
Buse 2004 [92]	ExBID+MET +/or SFU	51	13	36	No	
	5 mcg ExBID+MET +/or SFU	39	10	14	No	
	PBO+MET +/or SFU	7	2	3	No	
Buse 2007 [93]	ExBID+MET +/or SFU	8-39	NG	<1-13	0.2%	1 severe hypo in 1010 subj-y
Davies 2009 [94]	ExBID+MET+/or SFU+/or TZD	48.3	NG	50.0	4.2%	Nocturnal hypo: 11.9%.
	Glarg+MET+/or SFU+/or TZD	2.6	NG	59.6	5.3%	Nocturnal hypo: 29.8%. P=0.001 vs Ex
DeFronzo 2005 [95]	ExBID+MET	45	12	5	No	
	5 mcg ExBID+MET	36	11	5	No	
	PBO+MET	23	4	5	No	
Derosa 2010 [96]	ExBID+MET	NG	NG	0	NG	
	Glibenclamide+MET	NG	NG	4.6	NG	
Derosa 2011 [97]	ExBID+MET	NG	NG	0	NG	
	Glibenclamide+MET	NG	NG	5.6	NG	
Forti 2008 [98]	ExBID at B/D+MET± SFU±TZD	22.6	10.0	NG	4.2%	5.21 hypo/subj-y. P=0.393 vs Ex L/D
	ExBID at L/D+MET± SFU± TZD	25.1	10.2	NG	0.5%	6.51 hypo/subj-y
Gallwitz 2011 [99]	ExBID+MET	18.8	9.9	8.0	No	Nocturnal hypo: 3.9%
	Aspart+MET	NG	NG	20.5	No	Nocturnal hypo: 7.0%
Gao 2009 [100]	ExBID+MET±SFU	25.5	15.8	35.5	0.9%	4.4 hypo/subj-y. MET only: 1.8 hypo/subj-y MET+SFU: 4.7 hypo/subj-y
	PBO+MET±SFU	0.9	0	9.0	0.4%	0.5 hypo/subj-y MET only: 0.22 hypo/subj-y MET+SFU: 0.54 hypo/subj-y
Gill 2010 [101]	ExBID+MET +/or TZD	36	NG	7	No	
	PBO+MET +/or TZD	19	NG	4	No	
Heine 2005 [102]	ExBID+MET+SFU	57.1	17.4	NG	1.4%	7.3 hypo/subj-y. Nocturnal hypo: 21%; 0.9 hypo/subj-y
	Glarg+MET+SFU	8.6	3.7	NG	1.5%	6.3 hypo/subj-y. Nocturnal hypo: 43%; 2.4 hypo/subj-y
Kadowaki 2009 [103]	ExBID+SFU±MET, +SFU±TZD	35.1	8.1	54.1	No	
	5 mcg ExBID+SFU±MET, +SFU±TZD	8.1	13.5	43.2	No	
	2.5 mcg ExBID+SFU± MET, +SFU±TZD	10.8	5.4	27.0	No	
	PBO+SFU±MET, +SFU±TZD	0	0	10.0	No	
Kadowaki 2011 [104]	ExBID+SFU, +SFU+MET, +SFU+TZD	36.1	16.7	58.3	No	
	5 mcg ExBID+SFU, +SFU+MET, +SFU+TZD	25.0	4.2	51.4	No	
	PBO+ExBID+SFU, +SFU+MET, +SFU+TZD	8.6	2.9	22.9	No	
Kendall 2005 [105]	ExBID+MET+SFU	48.5	13.7	27.8	No	Lower hypo incidence in SFU MIN group
	5 mcg ExBID+MET+SFU	39.2	14.7	19.2	4.1%	
	PBO+MET+SFU	20.6	4.5	12.6	No	
Klonoff 2008 [106]	ExBID+MET+SFU	59.0	NG	40.0	0.2%	All severe hypo events occurred in patients treated with SFU
Liukus 2010 [107]	ExBID+TZD±MET	12	8	4.0	No	
	PBO+TZD±MET	2	0	2.0	No	
Moretto 2008 [108]	ExBID	13	4	4	No	0.52 hypo/subj-y
	5 mcg ExBID	3	4	5	No	0.21 hypo/subj-y
	PBO	0	0	1	No	0.03 hypo/subj-y. P=0.014 vs Ex
Nauck 2007 [109]	ExBID+MET+SFU	33.2	15.0	NG	No	4.7±0.7 hypo/subj-y Nocturnal hypo: 17%.
	Aspart+MET+SFU	0.4	3.2	NG	No	5.6±0.7 hypo/subj-y Nocturnal hypo: 25%; P<0.038 vs Ex
Ratner 2006 [110]	ExBID+MET	14-33	1-5	NG	No	
Riddle 2006 [111]	ExBID+SFU±MET	14-27	NG	8-15	1.0%	
Zinman 2007 [112]	ExBID+TZD±MET	39.7	13.2	10.7	No	
	PBO+TZD±MET	15.2	0.9	7.1	No	
**EXENATIDE QW CLINICAL TRIALS**						
Bergenstal 2010 [113]	ExQW+MET	24	11	1	No	
	SITA+MET	10	2	3	No	
	PIO+MET	5	3	1	No	2 cases of pancreatitis
Blevins 2011 [114]	ExQW+D/E, MET, SFU, TZD or any combo	14.0	4.7	3.9	No	All hypo events occurred in patients treated with SFU 1 case pancreatitis: resolved while taking ExQW.
	ExBID+D/E, MET, SFU, TZD or any combo	35.0	8.9	3.3	No	All hypo events occurred in patients treated with SFU
Buse 2010 [115]	ExQW+D/E, MET, SFU, TZD or 2 OADs	7.0	6.3	NG	No	All hypo events occurred in patients treated with SFU Wk 30-52: 10.2% hypo
	ExBID then ExQW, +D/E, MET, SFU, TZD or 2 OADs	7.7	4.6	NG	No	All hypo events occurred in patients treated with SFU Wk 30-52: 8.0% hypo
Buse 2011^1^	ExQW+MET, SFU, TZD or any comb	9.3	3.7	10.8	No	
	LIRA+MET, SFU, TZD or any comb	20.4	10.7	8.9	No	
Diamant 2010 [116]	ExQW+MET±SFU	13	4	8	No	
	Glarg+MET±SFU	1	1	26	No	Hypo with MET: p<0.0001 vs Ex+MET Hypo with MET+SFU: p=0.0009 vs Ex+MET+SFU Nocturnal hypo Hypo with MET: p=0.028 vs Ex+MET Hypo with MET+SFU: p=0.0005 vs Ex+MET+SFU
Drucker 2008 [117]	ExQW+D/E, MET, SFU, TZD or 2 OADs	26.4	10.8	5.4	No	Hypo with SFU: 14.5% vs 0% without SFU
	ExBID then ExQW, +D/E, MET, SFU, TZD or 2 OADs	34.5	18.6	6.1	No	Hypo with SFU: 15.4% hypo vs 1.1% without SFU
MacConell 2011^2^	ExQW+D/E, MET, SFU, TZD or 2 OADs	16 (wks 30- 156)	NG	NG	No	
Russell-Jones 2012 [118]	ExQW	11.3	4.8	5.2	No	
	MET	6.9	3.3	4.1	No	
	PIO	4.3	3.1	3.7	No	
	SITA	3.7	1.8	3.1	No	
Taylor 2011 [119]	ExQW+D/E, MET, SFU, TZD or 2 OADs	12*	9*	6.6*	No	*Wk 30-104 Hypo with SFU: 94% (16/17 subj)
Wysham 2011 [120]	ExQW+MET	5.0**	5.0**	1**	No	**Wk 26-52
	SITA then ExQW, +MET	10.8**	3.8**	2**	No	**Wk 26-52
	PIO then ExQW, +MET	9.6**	2.6**	1**	No	**Wk 26-52

Note: when 'ExBID' is listed without dose, dose was 10 mcg BID. When ExQW listed without dose, dose was 2 mg.

Abbreviations: Aspart insulin aspart; B, breakfast; BID, twice daily; D, dinner; Ex, exenatide; GLARG, insulin glargine; Hypo, hypoglycemic events; hypo/subj-y, number of hypoglycemic events per subject year; L, lunch; MET, metformin; MIN, minimum; Lira, liraglutide; NG, not given; PBO, placebo; PIO, pioglitazone; QW, once weekly; Sita, sitagliptin; Subj, subject; SFU, sulfonylurea; TZD, thiazolidinedione; vs, versus; Wk, week; Y, year.

## Footnotes

1 Buse JB, Nauck MA, Forst T, *et al.* Efficacy and safety of exenatide once weekly versus liraglutide in subjects with type 2 diabetes (DURATION-6): a randomised, open-label study. Diabetologia 2011; 54(Suppl 1): S38.

2 MacConell L, Walsh B, Li Y, Pencek R, Maggs D. Exenatide once weekly: sustained improvement in glycemic control and weight loss through 3 years. Diabetes 2011; 60: 969-P.

3 MacConell L, Gurney K, Malloy J, Zhang H, Zhou M, Chen S. Safety and tolerability of exenatide once weekly in patients with type 2 diabetes: an integrated analysis of 4328 patients. Diabetes 2012; 61(Suppl 1): A598 (2372-PO).

4 MacConell L, Malloy J, Huang W, Cirincione B, Shen L, Porter L. Safety and efficacy of once-monthly exenatide over 20 weeks in patients with type 2 diabetes. Annual meeting of the American Diabetes Association, June 2011. Late-breaking abstract 46-LB accessed at http://professional.diabetes.org/Congress_Display.aspx?TYP=9&CID=82452

5 Exenatide study of cardiovascular event lowering trial (EXSCEL): a trial to evaluate cardiovascular outcomes after treatment with exenatide once weekly in patients with type 2 diabetes mellitus. NCT01144338. Accessed May 14, 2012 at http://clinicaltrials.gov/ct2/show/NCT01144338

## ABBREVIATIONS

AERS: = adverse event reporting system
ALT: = alanine aminotransferase
ApoB: = apolipoprotein B
ASPART: = biphasic insulin aspart 70/30 mix
AST: = aspartate aminotransferase
B: = breakfast
BG: = background
BG: = background
BID: = twice daily
BL: = baseline
BP: = blood pressure
BIM: = body mass index
Comb: = combination
CV: = cardiovascular
CVD: = cardiovascular disease
D: = dinner
D/E: = diet and exercise
DO: = study dropout rate
DPP-4: = dipeptidyl peptidase-IV
Dur: = duration of clinical trial
DURATION: = Diabetes therapy Utilization Researching changes in HbA_1c_, weight and other factors Through Intervention with exenatide ONce weekly
EAIR: = Exposure-adjusted incidence rate
Ex: = exenatide
GLP-1: = glucagon-like peptide-1
EASD: = European Association for the Study of Diabetes
F: = female
FDA: = U.S. Food and Drug Administration
GIP: = glucose-dependent insulinotrophic polypeptide/ gastric inhibitory polypeptide
Geo: = geometric
GLARG: = insulin glargine
HDL-C: = high density lipoprotein cholesterol
Gli: = glibenclamide sulfonylurea
HbA_1c_: = glycated hemoglobin
HOMA: = homeostasis model assessment
hsCRP: = high sensitivity C-reactive protein
Hypo: = hypoglycemic events
ILM: = intensive lifestyle modification
KO: = knockout
L: = lunch
LDL-C: = low density lipoprotein cholesterol
LIRA: = liraglutide
LS: = least squares
MACE: = major adverse cardiac events
MET: = metformin
MIN: = minimum
NA: = not applicable
NG: = not given
NI: = non-inferiority
OAD: = oral antidiabetes drug
PBO: = placebo
PIO: = pioglitazone
Pop: = population
PTH: = parathyroid hormone
QD: = once daily
QM: = once monthly
QW: = once weekly
oxLDL: = oxidated low density lipoprotein
ROSI: = rosiglitazone
ΔRx: = change or different diabetes medication prior to randomization
S: = superiority
SFU: = sulfonylurea
Sita: = sitagliptin
Sita: = sitagliptin
Subj: = subject
T2DM: = type 2 diabetes mellitus
T_50_: = time to achieve 50% stomach emptying
TID: = thrice daily
T_max_: = time to reach maximal blood concentration
TSH: = thyroid stimulatory hormone
TZD: = thiazolidinediones (TZD)
VLDL-C: = very low density lipoprotein cholesterol
vs: = versus
Wk: = week
Wt: = weight
Y: = year

## Clinical Trial Registration Numbers:

See Table **1**
